# DNA Length-dependent Division of a Giant Vesicle-based Model Protocell

**DOI:** 10.1038/s41598-019-43367-4

**Published:** 2019-05-06

**Authors:** Muneyuki Matsuo, Yumi Kan, Kensuke Kurihara, Takehiro Jimbo, Masayuki Imai, Taro Toyota, Yuiko Hirata, Kentaro Suzuki, Tadashi Sugawara

**Affiliations:** 1 Department of Basic Science, Graduate School of Arts and Sciences, The University of Tokyo, Komaba, Meguro, Tokyo 153-8902 Japan; 2 Department of Creative Research, Exploratory Research Center on Life and Living Systems (ExCELLS), Myodaiji, Okazaki, Aichi 444-8787 Japan; 3 Department of Physics, Graduate School of Science, Ochanomizu University, Otsuka, Bunkyo, Tokyo 112-8610 Japan; 4 Determent of Life and Coordination-Complex Molecular Science, Biomolecular Functions, Institute for Molecular Science, Myodaiji, Okazaki, Aichi 444-8585 Japan; 5 Department of Physics, Graduate School of Science, Tohoku University, Aoba, Sendai, Miyagi 980-8578 Japan; 6 Universal Biology Institute, The University of Tokyo, Hongo, Bunkyo, Tokyo 113-0033 Japan; 7 Department of Chemistry, Faculty of Science, Kanagawa University, Tsuchiya, Hiratsuka, Kanagawa 259-1293 Japan

**Keywords:** Emergence, Synthetic biology, Origin of life, Supramolecular chemistry

## Abstract

DNA is an essential carrier of sequence-based genetic information for all life today. However, the chemical and physical properties of DNA may also affect the structure and dynamics of a vesicle-based model protocell in which it is encapsulated. To test these effects, we constructed a polyethylene glycol-grafted giant vesicle system capable of undergoing growth and division. The system incorporates a specific interaction between DNA and lipophilic catalysts as well as components of PCR. We found that vesicle division depends on the length of the encapsulated DNA, and the self-assembly of an internal supramolecular catalyst possibly leads to the direct causal relationship between DNA length and the capacity of the vesicle to self-reproduce. These results may help elucidate how nucleic acids could have functioned in the division of prebiotic protocells.

## Introduction

Giant vesicle (GV)-based model protocells (diameter >1 µm) have recently been constructed for investigating the intrinsic nature of cellular life^1–7^. One advantage of using GV-based model protocells in this context is the structural simplicity of the GV and its components, which facilitates examination of specific interactions among three indispensable elements, e.g., lipids forming a compartment, RNA/DNA carrying information, and proteins working as enzymes. Studies on some aspects of primitive cellular life exhibited by GV-based model protocells with the same RNA/DNA sequence-based genetic system as that carried by contemporary cells have been reported; these aspects have included protein expression^8^, molecular evolution^9^ and membrane lipid biosynthesis^10–12^, although no linked proliferation between DNA replication and GV reproduction was exhibited in these model protocells.

**Figure 1:**
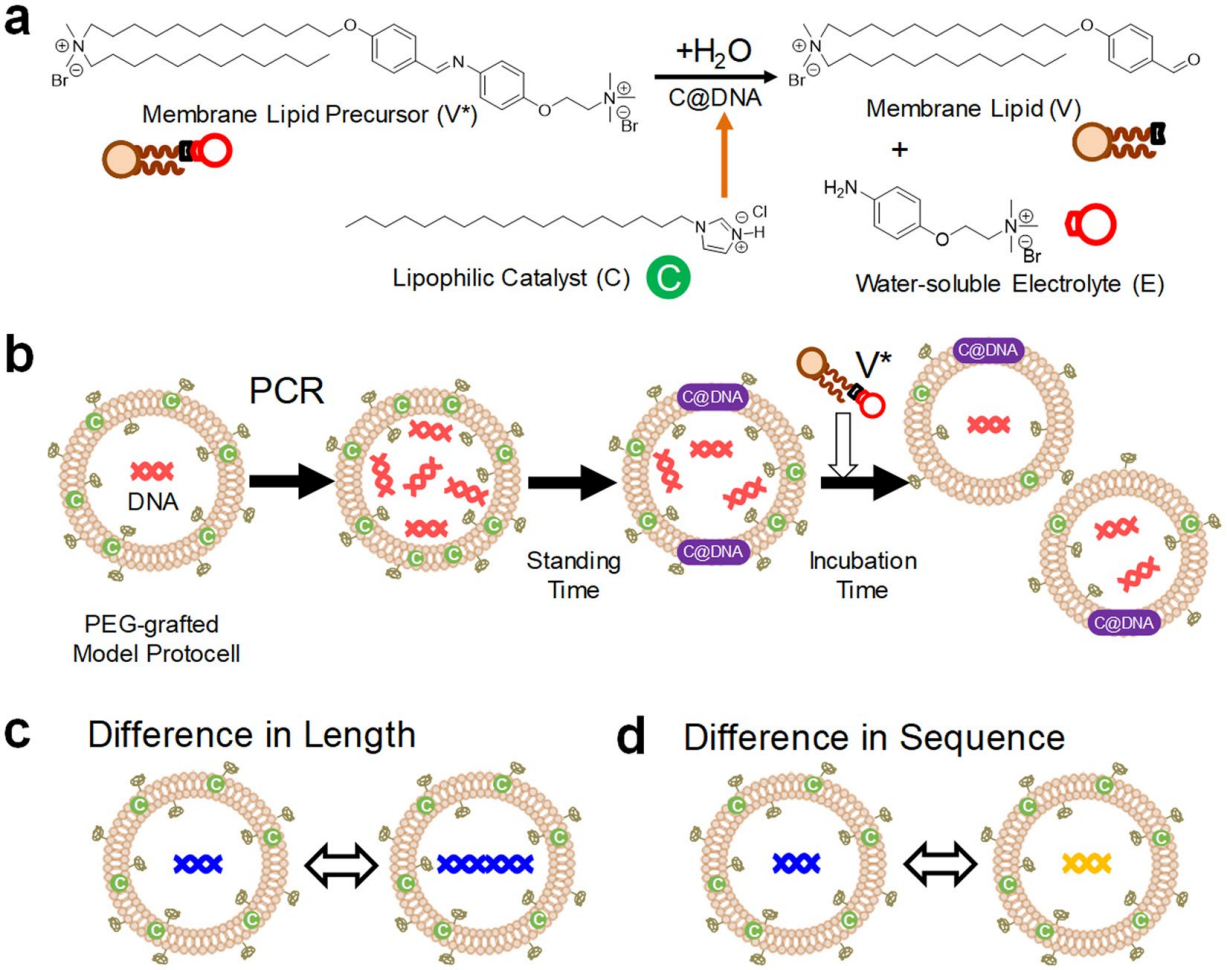
Schematic illustration of the self-proliferating GV-based model protocell and dependence of its division on the characteristics of the encapsulated DNA. Chemical structures of the constituents of a GV-based model protocell (**a**). Self-proliferating PEG-grafted model protocell (**b**). PEG-grafted model protocells containing DNA of different lengths (**c**). Model protocells containing DNA with different sequences (**d**).

Since DNA replication and GV reproduction are independent events, it has been considered difficult to couple these two dynamics; however, their linkage has already been achieved^13,14^. A self-reproducing GV has been characterized as follows (Fig. 1a,b). When a membrane precursor (V*) was added to a dispersion of our self-reproducing GVs, the precursor V* dissolved in the GV membrane and was hydrolyzed to form a membrane lipid (**V**) and an electrolyte (**E**). Since the electrolyte **E** easily dissolved in water, the equilibrium greatly shifted toward the product side. The increased amount of membrane lipids **V** in the GV membrane put pressure on the surrounding membrane lipids **V** and deformed the GV, which eventually divided into two GVs. The optimal size of a GV is regulated by the elasticity of the membrane, which is derived from the spontaneous curvature originating from a packing parameter that is based on the shape of the amphiphile^15^. A linkage between the self-reproduction of a GV and the amplification of the DNA in a GV was revealed after adding cationic membrane lipids **V** to the vesicular membrane, the main components of which were phospholipids. If this GV contains PCR reagents and is subjected to PCR, the amplified DNA accelerates the division of GV, working cooperatively with cationic lipids (**C**) to produce membrane lipids **V** from the precursor of membrane lipids V*. To transfer the linked self-production ability to daughter GVs, the DNA in the original GV must be amplified before the division. PCR is a useful method to amplify the DNA because the denaturation of double-stranded DNA is achieved by thermal energy and DNA polymerase as the sole protein.

In our model protocells, the physical and chemical properties of DNA may also affect the structure and dynamics of the system in which it is encapsulated. To test this possibility, we investigated the effect of encapsulating DNA with different properties in our model protocell on the manner of GV division, focusing on the interaction between the encapsulated DNA and vesicular membrane.

Even in the prebiotic era, a physical and chemical causal effect could have existed before the primitive flow of genetic information. We are interested in uncovering the emergence of such a physical and chemical causal effect. This study presents the development of a chemical system that should be able to demonstrate the potential for inheritance over generations without relying on a direct transcription and translation (TX-TL) system.

## Results and Discussion

### Construction of a PEG-grafted GV as a model protocell

Modification of bioinspired membranes is often observed, e.g., a phospholipid bearing a polymerizable group and an artificial signal transduction system^16,17^. In this study, we modified the composition of a vesicular membrane by adding polyethylene glycol (PEG)-grafted phospholipids (DSPE-PEG1000, the corresponding chemical nomenclature is described in the Methods section, (Materials); the same for terms hereinafter) to strengthen the subtle differences in division in response to encapsulated DNA with different properties. The coverage of the vesicular membrane surface by PEG-grafted phospholipids regulates the interaction between DNA and the positively charged membrane surface (Fig. S1).

PEG chains grafted onto phospholipids form mushroom-like structures at the interface between the water phase and the vesicular membrane (Fig. S1), and these structures suppress the interaction between DNA and cationic lipids (**V** and **C**) via not only steric repulsion but also the weak solvent effect of DNA^18–20^. The molar fractions of the PEG-grafted phospholipids used in the present study were ca. 0.9 mol%, as previously reported^21^, which corresponds to coverage of approximately one-quarter of the whole inner surface of the GV (Eq. S6)^22^. Eventually, we prepared PEG-grafted GVs containing 1164 bp DNA and with a membrane composed of POPC:**V**:**C**:cholesterol:DSPE-PEG1000 (mol%: 78:4.2:8.5:8.5:0.9); the GVs were stained with Texas Red-DHPE (0.2 mol%). The PEG chain of DSPE-PEG5000 was also used to modify the membrane, but almost no divisions were observed after the addition of V* probably because PEG5000 completely suppressed the interaction between DNA and GV membranes containing cationic lipids (Table S1).

### Effect of DNA of different lengths studied by population analysis

**Figure 2:**
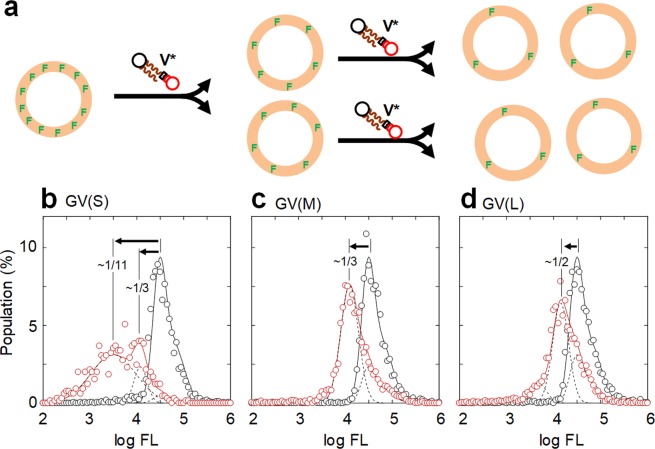
Histograms of the fluorescence intensities of BODIPY-stained GVs (S), GVs (M), and GVs (L) before and after V* addition. Schematic illustration of the self-proliferation of a fluorescence dye-stained GV. The amount of fluorescent dye decreases with each division (**a**). Population changes of BODIPY-stained GVs (S) (**b**), GVs (M) (**c**), and GVs (L) (**d**) induced by the addition of V* with a standing period of 48 h: before (black line) and at 40 min after (red line) V* addition. The histogram profiles were deconvoluted by Gaussian curves (dotted lines).

It is firmly established that DNA sequence information is key to the current gene expression system. Because this GV-based model protocell is not equipped with a TX-TL system, we focused on the effect of DNA length on the membrane dynamics of model protocells (Fig. 1c). To investigate each self-proliferating GV-based model protocell containing DNA of different lengths, we first used flow cytometry (FCM) to analyze the changes in GV populations. FCM allows for the exploration of the distribution of a large number of vesicles (10^4^) in reference to a size standard or to the fluorescence intensity emitted by a fluorescent probe attached as a tag to a vesicle component. For the FCM measurements, we prepared GVs containing short 374 bp DNA [GV (S)], medium-length 1164 bp DNA [GV (M)], and long 3200 bp DNA [GV (L)] (sequences given in Fig. S2) using the membrane compositions mentioned above and a solution of PCR reagents that included different DNA for rehydrating the membrane lipids (Table S2). The vesicular membranes were stained with BODIPY-HPC (0.1 mol%). A decrease in the fluorescence intensity emitted from the membrane containing the fluorescence probe reflects division of the GVs. Accordingly, the population change in the histogram of the fluorescence intensities corresponds to the frequency of division (Fig. 2a)^23,24^.

A histogram of the fluorescence intensities of PCR-subjected GVs with a standing period of 48 h was generated after incubation for 40 min following the addition of V* and a marked change in the distribution of the histogram is shown in Fig. 2. For GV (S), peaks centered at 4.5 and 3.5 (logarithmic value of the fluorescence intensity, arbitrary unit), which correspond to the original and new GV peaks, respectively, were detected. These peaks could be formed by budding deformation, followed by division (this type of division is defined as normal division). In addition to these peaks, a very broad peak corresponding to much lower fluorescence intensities was present (not shown in Fig. 2). Since the self-reproduction of GVs occurred several times at most in an hour, this broad peak must primarily correspond to abnormal decompositions of GVs not directly related to normal division. If we plotted only GVs with diameters of 5 μm or higher, the broad peak was absent. GVs with diameters less than 5 μm were detected by measuring the forward-scattered light intensity in the FCM data; the scattered light intensity was converted to the diameter of a GV using beads with known diameters (Eq. S7). Consequently, a factor related to the FCM measurement places a size limit of 5 μm or higher for a self-reproducing model protocell, which is a reasonable limit. Generally, enzymatic reactions can proceed within GVs of a broad range of sizes^21^.

The fluorescence intensity histogram of GV (S) shows that almost all the original population corresponding to the peak at 4.5 has disappeared (only 5% of the area intensity remaining) and new populations producing peaks at 3.5 and 4.0 appeared (95% of the original intensity) (Fig. 2b). These results indicate that most GVs (S) divided a few times or more within 40 min, producing many GVs smaller than the original GVs (Fig. S4). The new populations of GVs (M) and GVs (L), corresponding to peaks 4.1 and 4.2, constituted 75% and 67% of the original populations and resulted in reductions of one-third and one-half in the fluorescence intensities, respectively, as determined from the shifts along the horizontal axis (Fig. 2c,d). The population reduction of GVs (L) was slightly smaller than that of GVs (M). Interestingly, the population change during a standing period of 30 min after PCR was almost the same for GVs (S) and GVs (M), whereas that for GVs (L) was only 60%, suggesting a slower division rate for GVs (L) (Table S3). As shown by the histogram of the fluorescence intensities, the population changes of the three kinds of GVs depended on the difference in DNA length. The division of GVs (S) was fast but yielded only small GVs, while GVs (M) produced a reasonable number of daughter GVs with sizes almost equal to that of the mother GV (equivolume division is defined as when the volume of a divided daughter GV is not less than 70% the size of the mother GV).

### Counting increased numbers of GVs by wide-view/high-precision confocal microscopy

**Figure 3:**
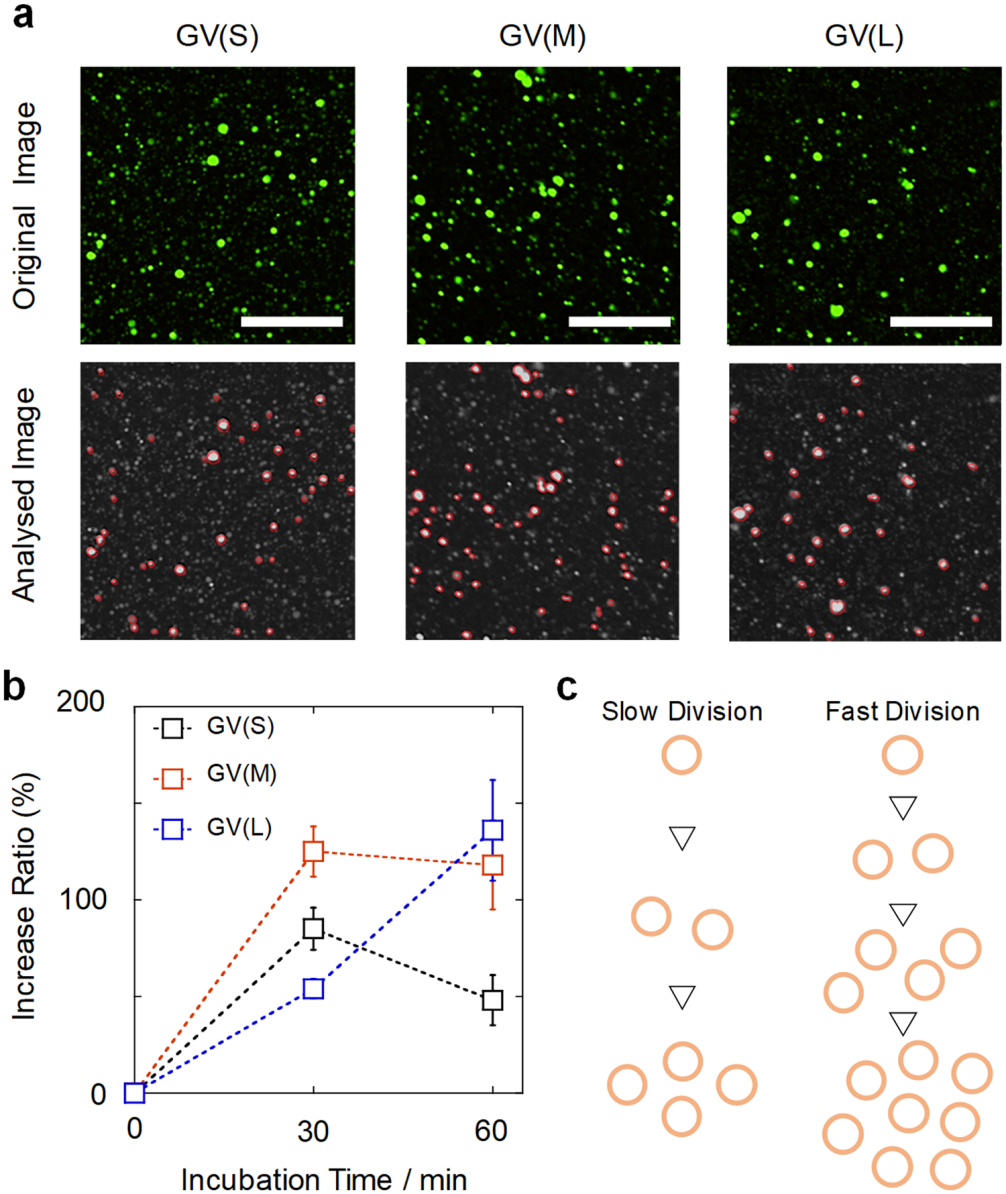
Confocal microscopy sliced images showing the increase in GVs containing different types of DNA and dependence of the percent increase on the incubation period for GVs (S), (M), and (L). Representative expanded original images (338 µm × 338 µm) of GVs (standing period of 48 h) at 30 min after the addition of V* (**a**, above); *z*-stacks were used to process the images. Scale bars represent 125 µm. GVs with diameters ≥5 µm are marked by red lines, while GVs with diameters <5 µm are not marked (**a**, bottom). Plot of the dependence of the percent increase on the incubation period for GVs (S), (M), and (L). Error bars represent the standard errors (**b**). Schematic illustration showing different increases in GVs containing different kinds of DNA, reflecting the difference in the rate of division (**c**).

An increased number of GVs is the most straightforward reflection of the correlation between the characteristics of the encapsulated DNA and the ability to undergo division. Therefore, we directly counted the number of GVs (10^2^–10^3^ GVs, diameter ≥5 μm) before and after the addition of V* under a wide-view/high-precision confocal laser-scanning fluorescence microscope. In practice, after PCR and 48 h of incubation, a dispersion of GVs (1 mM) was diluted two-fold by mixing with a V* solution (1 mM) in a volume-to-volume ratio of 1:1. Then, the mixture was immediately placed into a frame chamber (9 mm × 9 mm, 25 µL) on a confocal microscope. GVs containing DNA in the observation field (1350 µm × 1350 µm) were captured as a sliced wide image (Figs 3a (above) and S5). The percent increase was calculated from the wide microscopy images for statistical analysis. The minimum diameter of countable GVs was technically 5 µm depending on the resolution of wide images under the observation condition. Therefore, the total number of GVs with diameters equal to or greater than 5 µm captured in 125 sliced wide images were counted by viewer and analyzer software (Figs 3a (below) and S6). The percent increase of GVs was defined as:

1 $${\rm{Increase}}\,( \% )=(\frac{2\times {\rm{Number}}\,{\rm{of}}\,{\rm{GVs}}\,{\rm{after}}\,{\rm{the}}\,{\rm{addition}}\,{{\bf{V}}}^{\ast }}{\mathrm{Number}\,\mathrm{of}\,\mathrm{GVs}\,\mathrm{before}\,\mathrm{the}\,\mathrm{addition}\,{{\bf{V}}}^{\ast }}-1)\times 100$$

The dependence of the percent increase in GVs on the incubation period is depicted in Fig. 3b. The increase in GVs (S) was 85% at 30 min after the addition of V* but 48% at 1 h, presumably because the formed GVs divided into GVs less than 5 µm in size. In contrast, the increase in GVs (M) was 125% at 30 min, after which time saturation occurred. The increase in GVs (L) was only 54% at 30 min but 136% at 1 h, reaching almost the same value as that of GVs (M). The direct confocal microscopy GV counts clearly indicate that the percent increase depends on the length of encapsulated DNA in the GV. The dependence is in agreement with the FCM results.

### Single GV observation by confocal microscopy

**Figure 4:**
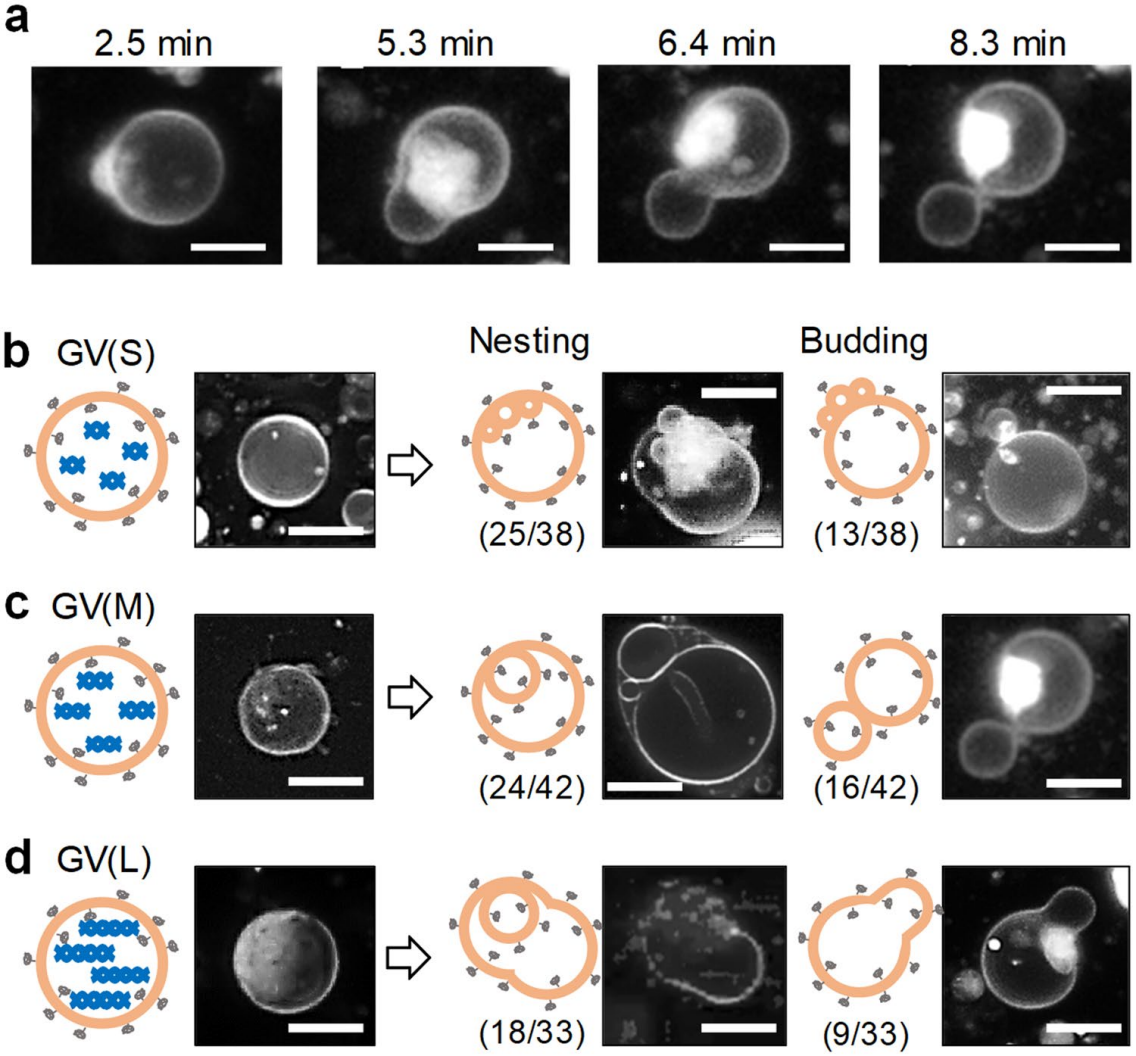
Morphological changes of GVs containing different kinds of DNA. Time-lapse images of the budding dynamics of a GV (M) (**a**). Representative confocal microscopy images of nesting and budding deformations of a GV (S), GV (M), and GV (L), together with schematic illustrations of the deformations (**b**–**d**). The relative ratios of each pattern are shown by *m*/*n*, where *m* indicates the number of deformed GVs and *n* is a number of specimens observed.

As shown by the FCM results, GVs containing DNA divided rapidly soon after the addition of V*. Hence, it was difficult to observe the morphological changes prior to division. However, the manner and rate of GV division induced by the addition of V* were highly variable. Some GVs underwent fast growth and division, but others exhibited division rates that allowed the GV deformation dynamics to be traced to explore the mechanism behind the DNA length dependence of the division. Hence, we observed the behavior of a single GV (diameter ≥5 µm) with thin lamellar layers under a high-speed confocal laser-scanning fluorescence microscope. We monitored the morphological changes over 40 min after the addition of V* to the dispersion of GVs (S), (M), and (L), which were stained with Texas Red-DHPE (0.2 mol%) (Fig. 4). The wide-angle view recorded under the confocal laser-scanning fluorescence microscope revealed that a sizeable fraction of the GVs underwent morphological changes (Fig. S7). This finding means that the deformation of GVs after the addition of V* is not a rare event.

PEG-grafted GVs afforded nested GVs in relatively high ratios (Fig. 4). The nesting deformation was thought to be derived from an inner budding created by preferential formation of membrane lipid **V** in the inner leaflet due to the presence of PEG moieties, and the DNA simply adhered to the inner surface of the GV membrane. The reason for the higher amount of the nested GVs (S) than of GVs (M) and (L) may be as follows. A mild disturbance of the lipid membrane caused by the adhesion of short DNA prohibits the lipid-coating on the adhered DNA, while a significant disturbance caused by the adhesion of medium-length or long DNA allows these DNA pieces to be coated with the membrane lipid and induce budding deformation.

No equivolume budding was observed in GVs (S). 38% (16/42) of GVs (M) and 9% (9/33) of GVs (L) exhibited almost the equivolume budding (Table S4). This tendency must be derived from the increase in **V** within the outer leaflet, suggesting that more than one-third of the amplified DNA in GVs (M) interacted with the membrane lipids and created **C@DNA** there. On the other hand, the proportions of non-deformed GV at 40 min after the addition of V* were 0%, 5% and 18% for GV (S), GV (M), and GV (L), respectively. The reason that the proportion of non-deformed GV (L) was the largest may be derived from the slow deformation rate of GV (L) due to the following two reasons (Table S4). First, long DNA was difficult to be coated entirely, but interacted only partially with membrane lipids. Second, the membrane of GV (L) was lined with long pieces of DNA and became hard to bend. It may be argued that the division of a budded GV is different from the birthing where a new GV is extruded from the nested GV. However, these two dynamics, division and birthing, are designated “division” in this text in a broad sense because the number of GVs increases in both cases.

The real-time observation of self-proliferating GVs with different types of DNA revealed the process underlying the DNA-length-dependent morphological changes, and the result provided an explanation for the differences in the percent increase of GVs with different lengths of DNA.

### Effect of different DNA sequences

We observed that different lengths of DNA with specific sequences resulted in different percent increases in GVs, and it was also necessary to determine whether differences in the DNA sequence itself cause an increase when the length is similar. Hence, we prepared DNA with lengths of 1137 bp and 1192 bp, the sequences of which were different from that of medium-length DNA (1164 bp) used in the previous experiments (Fig. 1d). These DNA fragments (1137 bp and 1192 bp) were excised from the same vector pBR322, and the sequence of a third fragment (1164 bp) was random except for the primer-recognition region (Supplementary Note 3). The percent increases of the GVs containing the four kinds of DNA with different sequences were practically the same (Table S5). Therefore, the sequences are not used as information in the current GVs, i.e., the TX-TL system is not involved.

### Formation and function of C@DNA as a supramolecular catalyst

To understand the mechanism behind the correlation of DNA length and manner of division in the GV-based model protocell, the interaction between the DNA and the cationic lipids of the membrane must be elucidated. For this experiment, we used the following membrane composition, which lacked PEG-grafted phospholipids: POPC:POPG:**V**:**C**:cholesterol (mol%) = 35:39:12:9.0:5.0. Since the positive charge of the GV membrane might interfere with DNA amplification by DNA polymerase in PCR, the anionic phospholipid POPG was used to balance the overall positive charge of the cationic membrane lipid **V**.

**Figure 5:**
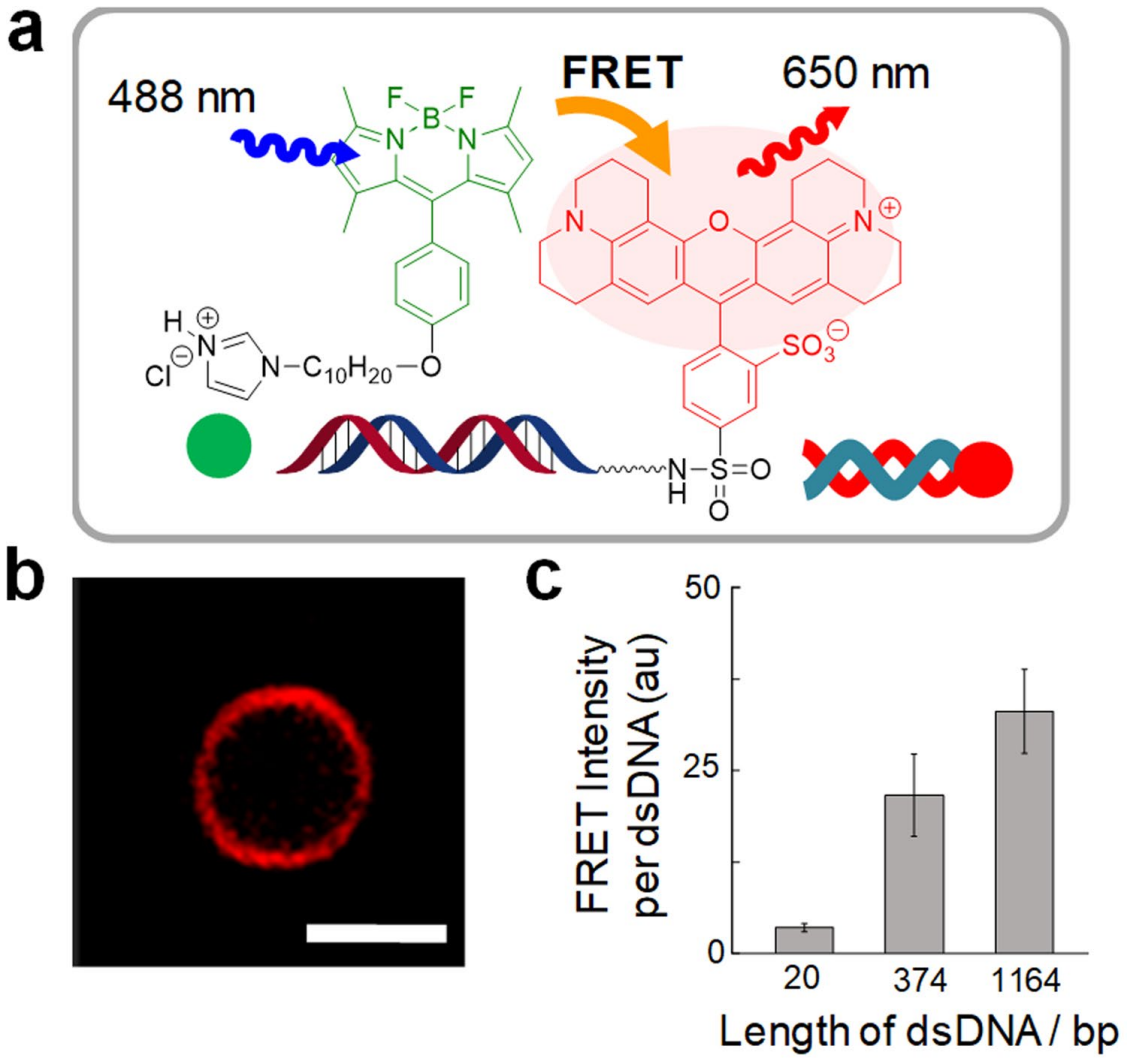
FRET from **C**-BODIPY to DNA-Texas Red and dependence of the FRET intensity on the length of DNA. Schematic of the FRET between **C**-BODIPY and DNA-Texas Red (**a**). Confocal microscopy image of a model protocell observed by the FRET channel (488 nm excitation, 650 nm emission) at 51 h after PCR. The scale bar represents 5 µm (**b**). Average fluorescence intensity (recorded by the FRET channel) emitted from the vesicular membrane (in the presence of 0.5 M TEMPOL in water) containing 20 bp (*n* = 6), 374 bp (*n* = 3) or 1164 bp (*n* = 5) DNA, respectively, at 51 h after the PCR; *n* is the number of examined specimens, and the error bars represent the standard errors (**c**).

We prepared DNA that was tagged with Texas Red (DNA-Texas Red) by amplifying template DNA in the presence of a Texas Red-tagged forward primer (Fig. 5a). First, to prove that DNA-Texas Red was coated with the vesicular membrane lipids and not simply adhered to the membrane’s hydrophilic surface, a hydrophilic fluorescence quencher was dissolved in the aqueous phase of a GV^25^. The red fluorescence from the vesicular membrane was not quenched in the presence of TEMPOL, which suggests that the DNA had been coated with the membrane lipids (Fig. S8). Next, to confirm that the DNA and **C** were located in close proximity, interacting with the membrane lipids, we utilized the Förster resonance energy transfer (FRET) method, where the excited energy transfer between two fluorescent dyes relates to their proximity. In this method, an energy donor and acceptor are needed; therefore, we prepared **C** tagged with the fluorescent dye BODIPY (**C-**BODIPY) as the energy donor and used DNA-Texas Red as the energy acceptor (Fig. 5a)^26,27^. FRET from **C**-BODIPY to DNA-Texas Red was clearly observed as intense red fluorescence emitted from the GV membrane (without TEMPOL; Figs 5b and S9), suggesting that **C-**BODIPY was located in close proximity to DNA-Texas Red in the vesicular membrane. Moreover, we prepared PCR-subjected GVs in which almost equal amounts of the three types of DNA (1.6 × 10^−18^ mol of 20 bp and 374 bp DNA and 0.90 × 10^−18^ mol of 1164 bp DNA) were encapsulated separately and found that the FRET intensity showed a clear dependence on the length of encapsulated DNA (Figs 5c and S10), which means that **C** is localized around the DNA in the vesicular membrane^28–30^. The FRET emitted from the membrane of PEG-grafted GVs was also observed regardless of the presence of a PEG chain, which is supposed to suppress the adhesion of DNA to the vesicular membrane (Fig. S11). The coating of the membrane lipids on DNA and the localization of lipophilic **C** around DNA indicate the formation of a complex of DNA with **C** (**C@DNA**).

**Figure 6:**
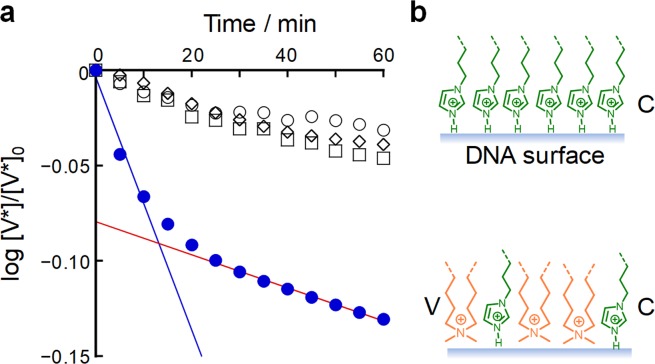
Synergistic catalytic effect of **C** and DNA. Time course of the decay rate of the membrane precursor V* (log ([V*]/[V*]_0_) in the presence of a dispersion of both DNA and **C** with lipid membranes (blue circles), a solution of DNA only (white squares), a dispersion of **C** and lipid membranes only (white diamond), and buffer only (white circles); these concentrations were 16 times lower than those used for the microscopy observations (**a**). DNA acts as a cocatalyst in **C@DNA**. Catalytic activity decreased when preoccupied **C** was replaced by generated **V** (**b**).

To study the catalytic activity of aggregates of **C@DNA** with membrane lipids for the hydrolysis of the membrane precursor V* to a membrane lipid **V**, we examined the rate of hydrolysis of V* over time in a buffered solution of such aggregates (Fig. S12)^31,32^. The hydrolysis of V* was analyzed in terms of pseudo-first-order kinetics (Fig. 6a). The rate of decay was faster in the presence of the **C@DNA** aggregates (half-life = 0.76 h) than in the presence of DNA solution or lipids containing only **C** (half-life = 7.2 h). This result clearly shows that the DNA increases the catalytic activity of **C** for the hydrolysis of V*, which means that the DNA acts as a cocatalyst. However, the rate of the initial fast decay in the presence of the **C@DNA** aggregates slowed down and it became comparable to the rate observed in the presence of DNA or **C** only, suggesting that the suppression of the synergetic effect is possibly due to the displacement of **C** from the complex, accompanied by the production of **V** (Fig. 6b).

The above results strongly suggest that **C@DNA** acts as a supramolecular catalyst in which catalysts **C** are arranged along the DNA chain through a self-assembly process (Fig. S13). The enhanced catalytic activity of **C@DNA** may resemble that of cyclodextrin bis(imidazole), which was elaborately designed as an artificial enzyme^33^. **C@DNA** does not manipulate the DNA but catalyzes the hydrolysis of V* by cleaving the imine bond, similar to the action of ribozymes—the RNA forms a complex with a metal ion and is able to catalyze the hydrolysis of RNA and other substrates^34–36^. In this sense, the supramolecular catalytic **C@DNA** in which DNA is covered with the lipophilic catalyst **C** can be regarded as a “lipo-deoxyribozyme”.

## Conclusion

Genetic inheritance is crucial to the transmission of a trait over generations. However, we did not study genetic inheritance in depth because the DNA used was in a sequence-independent stage in this model protocell. Regarding the inheritance of the self-reproduction ability of the GV-based model protocell, daughter GVs that received DNA of the same length from a mother GV were able to produce granddaughters of similar size even after dNTP depletion if the daughters were supplied the depleted dNTP through intervesicular transport and then amplified DNA using the received DNA as a template, as discussed in the recent report^14^.

The current system suggests that DNA length can carry biological information as a simple polymer independent of its sequence and that this information directly influences the division of GVs as a trait. This ability will be transmitted to a daughter GV as long as the entire length of the DNA is transferred intact from a parent model protocell without relying on the TX-TL system. The currently established ‘genotype and phenotype’ relationship, the well-known central dogma, may have originated from such a physical and chemical cause and effect in the prebiotic era.

## Methods

### Materials

1-Palmitoyl-2-oleoyl-*sn*-glycero-3-phosphocholine (POPC), 1-palmitoyl-2-oleoyl-*sn*-glycero-3-phospho-(1-rac-glycerol) sodium salt (POPG), and 1,2-distearoyl-*sn*-glycero-3-phosphoethanolamine-*N*-[methoxy(polyethylene glycol)-1000/5000] (DSPE-PEG1000/5000) were purchased from Avanti Polar Lipids, Inc. (Alabaster, AL, USA). Cholesterol and 4-hydroxyl-TEMPO (TEMPOL) were purchased from Wako Pure Chemical Industries (Osaka, Japan). The membrane lipid molecule **V**, the amphiphilic catalyst **C**, the catalyst **C** tagged with BODIPY (**C**-BODIPY) and the membrane lipid precursor V* were prepared as previously reported [8, 9]. The restriction enzyme EcoRI, the pBR322 Vector Wizard® SV gel and the PCR clean-up system for purification were purchased from Promega Corporation (Madison, WI, USA). Primers and a primer tagged with Texas Red were purchased from Sigma-Aldrich (St. Louis, MO, USA). Random 1164 bp DNA was purchased from Fasmac (Kanagawa, Japan). Sequences of template DNA and primers are shown in Supplementary Note 3 and Method 2. DNA ladder markers and DNase I were purchased from Takara Bio Inc. (Shiga, Japan). KOD-Plus DNA polymerase was purchased from Toyobo Co., Ltd. (Osaka, Japan). SYBR® Green I was purchased from Lonza (Rockland, ME, USA). Texas Red 1,2-dihexadecanoyl-*sn*-glycero-3-phosphoethanolamine, triethylammonium salt (Texas Red-DHPE), 2-(4,4-difluoro-5,7-dimethyl-4-bora-3*a*,4*a*-diaza-*s*-indacene-3-dodecanoyl)-1-hexadecanoyl-*sn*-glycero-3-phosphocholine (BODIPY-HPC) and SYBR® Gold nucleic acid gel stain were purchased from Thermo Fisher Scientific Inc. (Waltham, Massachusetts, USA). TE-saturated phenol:chloroform:isoamyl alcohol (25:24:1, v/v/v) was purchased from Nacalai Tesque, Inc. (Kyoto, Japan). The coprecipitating agent ethachinmate and sodium acetate (3 M, pH 5.2) for ethanol precipitation were purchased from Nippon Gene Co., Ltd. (Tokyo, Japan). Precast 12.5% poly(acrylamide)gel (e-PAGEL minigel, AE-6000 E-T12.5 L) was purchased from Atto Corporation (Tokyo, Japan).

### Preparation of template DNA

To obtain template DNA for PCR amplification, 1 μg of pBR322 vector DNA was incubated for 16 h at 37 °C in 20 μL of standard restriction digest buffer containing 5 units of *Eco*RI. After incubation, the digested pBR322 DNA was purified by using a clean-up system. Linear templates for incorporation into GVs (i.e., ‘short’ [374 bp, nucleotides 200–573]; ‘medium’ [1164 bp, 200–1363]; and ‘long’ [3200 bp, 200–3399], [1192 bp, 1733–2924] and [1137 bp, 2263–3399]) were excised and amplified from the digested pBR322 vector by using primer sets. The template fragment for short DNA was amplified by using forward primer 1 (5′-GACAGCATCGCCAGTCACTA-3′) and reverse primer 2 (5′-AATGGTGCATGCAAGGAGAT-3′); the template fragment for medium-length DNA was amplified by using forward primer 1 and reverse primer 3 (5′-TTTGCGCATTCACAGTTCTC-3′); the template fragment for long DNA was amplified by using forward primer 1 and reverse primer 4 (5′-CCCTCCCGTATCGTAGTTAT-3′); the template fragment for 1192 bp DNA was amplified by using forward primer 5 (5′-GGCATTGACCCTGAGTGATT-3′) and reverse primer 6 (5′-CTACATACCTCGCTCTGCTA-3′); the template fragment for 1137 bp DNA was amplified by using forward primer 7 (5′-TGCGGCATCAGAGCAGATTG-3′) and reverse primer 4; and the template for the ‘random-1164 bp’ fragment [sequence shown in Supplementary Note 3] was amplified by using forward primer 1 and reverse primer 3.

### General protocol for the preparation of the GV-based model protocell

GVs were prepared by freeze-drying as follows. Stock methanol solutions of the lipids were prepared independently and mixed in a test tube. The mixture was dried to a thin lipid film under reduced pressure to remove the solvent. The dry lipid film was hydrated by the addition of deionized water, followed by vortex mixing (30 sec), which resulted in the formation of a GV dispersion. The GV dispersion was incubated for 2 h at 25 °C, freeze-dried by rapid cooling in liquid nitrogen, and stored under reduced pressure over a period of 4.5 h. The obtained lipid powder was rehydrated with 500 µL of a PCR solution: deionized water (347 µL), PCR buffer (10× KOD-Plus buffer, 50 µL), MgSO_4_ aq. (25 mM, 20 µL), dNTP mixture solution (each 2 mM, 40 µL), forward primer aq. (10 µM, 14 µL), reverse primer aq. (10 µM, 14 µL), template DNA aq. (10 nM, 5 µL), and DNA polymerase aq. (0.6 μM, 1.0 U/μL KOD-Plus, Mg^2+^ free, 10 µL). To stabilize the resulting GVs, the dispersion was incubated for at least 1 h at 25 °C. After the incubation, to digest DNA in the exterior water phase of the GVs, buffer solution (500 µL) containing DNase I was added to the dispersion (total lipid concentration = 1 mM). The contents of the outer buffer solution were as follows: deionized water (435 µL), PCR buffer (10 × KOD-Plus buffer, 50 µL), CaCl_2_ aq. (100 mM, 5 µL), and DNase I (5,000 U/µL, 10 µL). The solution was incubated for 30 min at 25 °C.

### General protocol for DNA amplification by PCR

After incubation for 30 min at 25 °C, the GV dispersion was treated in a thermal cycler (iCycler, Bio-Rad Laboratories Japan Inc., Japan) for DNA amplification. The temperature program was as follows: 94 °C for 2 min and [94 °C for 15 sec and 68 °C for 90 sec] × 20 cycles. After the thermal cycles, the dispersion was slowly cooled to 25 °C.

### Polyacrylamide gel electrophoresis (PAGE)

Amplified DNA in the GVs or bulk solutions was purified by using a Wizard^®^ SV gel and PCR clean-up system (Promega Japan, Japan). Fractions from each column were loaded into the wells of a poly(acrylamide) gel in an electrophoresis apparatus (AE-6530M/P with a myPower500 power supply [AE-8150], Atto, Japan), and the gel was run under conditions of 250 V and 20 mA for 60 min. After electrophoresis, the gel was placed on a UV transilluminator (TFM-30, UVP, CA, USA), and DNA bands were visualized by using SYBR^®^ Gold nucleic acid gel stain (302 nm excitation/510–550 nm emission).

### General protocol for the preparation of PEG-grafted model protocells

In the current study, a dispersion of PEG-grafted GVs, the lipid composition of which was POPC:**V**:**C**:cholesterol:DSPE-PEG1000 in a ratio of 78:4.2:8.5:8.5:0.9 (mol%), were prepared by the freeze-drying using PCR solutions containing the different template DNA: 1164 bp, 374 bp and 3200 bp. The compositions of the PCR solutions are shown in Table S2. Thereafter, these dispersions were subjected to PCR.

A solution of membrane precursor V* (100 μL, 1 mM) was prepared by dissolving V* in a mixture of deionized water (445 µL), PCR buffer (10 × KOD-Plus buffer, 50 µL) and CaCl_2_ aq. (100 mM, 5 µL). After ultrasonication for 10 min, the solution of V* (100 μL, 1 mM) was added to the PCR-subjected dispersion of PEG-grafted GVs (100 μL, total lipid concentration of 1 mM).

### Population tracing of PEG-grafted GVs containing DNA of different lengths

A dispersion of PEG-grafted GVs that were stained by BODIPY-HPC (0.1 mol%) was prepared by freeze-drying, and DNA was amplified by PCR. A 500 μL solution of V* was added to 500 μL of the dispersion of PEG-grafted GVs at 0.5 or 24 h after PCR, and the mixture was transferred to a flow cytometer (SH800, SONY, Tokyo, Japan). The intensities of scattered light and fluorescence were detected by the flow cytometer on a mass scale (counting 10,000 GVs, 488 nm excitation/500–550 nm emission).

### Counting increased numbers of GVs by confocal laser-scanning fluorescence microscopy

A dispersion of PEG-grafted GVs that were stained by BODIPY-HPC (0.1 mol%) was prepared by freeze-drying, and DNA was amplified by PCR. After a standing period, the dispersion of PEG-grafted GVs was mixed with a membrane precursor V* solution at a volume ratio of 1:1. Then, the mixture was immediately placed into a frame chamber (9 mm × 9 mm, 25 μL) on a glass plate with a cover glass. The GVs in five 1350 μm × 1350 μm *x*-*y* observation fields (4 corners and one in the middle) in the frame chamber were captured as an *x*-*y* sliced image using an inverted microscope (Eclipse Ti, Nikon, Tokyo, Japan) equipped with a confocal laser scanner unit (CSU-W1, Yokogawa Electric Corp., Tokyo, Japan) and an sCMOS camera unit (Zyla 4.2 plus, Andor Technology Ltd., Belfast, United Kingdom) with 2048 × 2048 active pixels (488 nm excitation/500–550 nm emission). Along the *z* axis, 25 *x*-*y* sliced images were captured with a *z*-interval of 10 μm to constitute a *z*-stack. All GVs on 125 sliced images (25 images × 5 *x*-*y* positions) were counted by viewer and analyzer software (NIS-Elements, Nikon, Tokyo, Japan). The percent increase was calculated from the counted numbers of GVs before and after the addition of V* and treated by the Wilcoxon rank-sum test, together with the Bonferroni correction, to reveal the difference at the 5% significance level.

### Confocal microscopy observation of morphological changes in PEG-grafted GVs

A dispersion of PEG-grafted GVs stained with Texas Red-DHPE (0.2 mol%) was prepared with the same procedure described above. After incubation, the dispersion of PEG-grafted GVs was mixed with a membrane precursor V* solution at a volume ratio of 1:1. Then, the mixture was immediately placed into the frame chamber on a glass plate with cover glass. The dynamics of the PEG-grafted GVs were captured by a confocal microscope (LSM 5 LIVE, Zeiss, Tokyo, Japan) with a scan speed of 900 frames per second for 40 min after the V* addition (532 nm excitation/560–585 nm emission).

### Preparation of GVs for FRET experiments

To prepare GVs containing 1164 bp or 374 bp DNA template tagged with Texas Red, freeze-dried powder of lipids composed of POPC:POPG:**V**:**C**:**C**-BODIPY:cholesterol (35:39:12:8:1:5, mol%) were hydrated with 500 µL of PCR solution composed of deionized water (115 µL), PCR buffer (10 × KOD-Plus buffer, 50 µL), MgSO_4_ aq. (25 mM, 20 µL), dNTP mixture solution (2 mM each, 250 µL), primer 1 or primer 1-Texas Red (100 µM, 15 µL for both 1164 and 374 bp), primer 2 (for 1164 bp) or primer 3 (for 374 bp) (100 µM, 15 µL), 1164 bp or 374 bp DNA template (10 nM, 25 µL), and DNA polymerase (1.0 U/μL, KOD-Plus, Mg^2+^ free, 10 µL). Texas Red-tagged 20 bp DNA was prepared by adding a complementary Texas Red-tagged primer to a dispersion containing a Texas Red-tagged primer to be hybridized. In the case of GVs containing 20 bp DNA tagged with Texas Red, the freeze-dried lipid powder was hydrated with 500 µL of a DNA solution composed of 50 µL of a PCR buffer solution (10 × KOD-Plus buffer), 20 µL of MgSO_4_ aq. (25 mM), 15 µL of primer 1 (10 µM), 15 µL of anti-primer 1 (10 µM) carrying a complementary base sequence to primer 1, and 10 µL of DNA polymerase (1.0 U/μL, KOD-Plus, Mg^2+^ free) in 390 µL of deionized water. All dispersions of GVs were incubated at 25 °C for 1 h after the addition of DNase I, and the resulting GV dispersion was subjected to thermal cycles for the amplification of template DNA or the annealing of primer 1 and anti-primer 1 in the case of the formation of 20 bp DNA.

### FRET measurements with DNA-Texas Red and C-BODIPY

FRET from a donor (**C**-BODIPY) to an acceptor (DNA-Texas Red) was measured by a confocal microscope (LSM 5 LIVE, Zeiss, Tokyo, Japan) using three separate channels: a donor channel (**C**-BODIPY, 488 nm excitation/505–530 nm emission), an acceptor channel (DNA-Texas Red, 532 nm excitation/560–585 nm emission) and a FRET channel (488 nm excitation/560–585 nm emission) under the same gain, exposure time and laser intensity. The fluorescence intensity from individual target probes on the vesicular membrane was evaluated from the averages of the maximum intensities of 8 line profiles crossing over the GVs. Fluorescence intensities were treated by the Wilcoxon rank-sum test together with the Bonferroni correction to reveal the difference at the 5% significance level.

### Preparation of DNA-lipid aggregates and decay rate measurements of V*

The decay of V* was monitored by a UV-Vis spectrometer under four different conditions in reference to the additives in the buffer solution (**C** and DNA, catalyst **C** only, DNA only, and buffer only). A positive control sample was prepared as follows. First, a 500 µL KOD-Plus buffer solution of DNA (500 pM), dNTPs (200 µM each) and PCR reagents was subjected to PCR. Second, thin films composed of lipids (POPC:POPG:**V**:**C**:cholesterol = 35:39:12:9.0:5.0 mol%) were swelled with the above PCR-subjected buffer solution. The mixed solution (total lipid concentration of 2.5 mM) was diluted 16 times with KOD-Plus buffer and incubated for more than 1 h. Third, prior to UV spectroscope measurement, V* was swiftly dissolved in the KOD-Plus buffer dispersion of GVs and DNA, and the resulting solution was added to the diluted dispersion of GVs. The final concentrations of lipids and V* for the UV measurement were both 156 μM. Three negative control samples (DNA only, catalyst **C** only, and buffer only) were prepared in a similar manner. A “DNA only” solution was prepared using lipid films without the catalyst **C**. The “catalyst only” solution was prepared using a buffer solution without DNA (nor dNTPs). The “buffer only” solution was prepared by dissolving only lipids into a buffer solution. Aside from the four solutions/dispersions, a V* solution was also prepared immediately before use. The decay of V* was monitored by measuring the decrease in the UV-Vis-absorption intensity at 332 nm, which is assigned to the benzylidene aniline moiety of V*, after mixing a V* solution with each sample solution, and the data were analyzed in terms of pseudo-first order kinetics.

## Supplementary information

Supplementary Information

## References

[1] Szostak JW, Bartel DP, Luisi PL (2001). Synthesizing life. Nature.

[2] Luisi, P. L. & Stano, P. *The minimal cell: The biophysics of cell compartment and the origin of cell functionality*. ch.11, pp.243–267 (Springer, 2011).

[3] Saha R, Chen I (2015). Origin of Life: Protocells Red in Tooth and Claw. Curr. Biol..

[4] Kurihara K, Matsuo M, Yamaguchi T, Sato S (2017). Synthetic Approach to biomolecular science by cyborg supramolecular chemistry. Biochim. Biophys. Acta.

[5] Walde P, Wick R, Fresta M, Mangone A, Luisi PL (1994). Autopoietic self-reproduction of fatty acid vesicles. J. Am. Chem. Soc..

[6] Oberholzer T, Wick R, Luisi PL, Biebricher CK (1995). Enzymatic RNA replication in self-reproducing vesicles: an approach to a minimal cell. Biochem. Biophys. Res. Comm..

[7] Mansy SS (2008). Template-directed synthesis of a genetic polymer in a model protocell. Nature.

[8] Noireaux V, Libchaber A (2004). A vesicle bioreactor as a step toward an artificial cell assembly. Proc. Natl. Acad. Sci. USA.

[9] Ichihashi N (2013). Darwinian evolution in a translation-coupled RNA replication system within a cell-like compartment. Nat. Commun..

[10] Kuruma Y, Ueda T (2015). The PURE system for the cell-free synthesis of membrane proteins. Nat. Protoc..

[11] Kuruma Y, Stano P, Ueda Y, Luisi PL (2009). A synthetic biology approach to the construction of membrane proteins in semi-synthetic minimal cells. Biochim. Biophys. Acta.

[12] Scott A (2016). Cell-free phospholipid biosynthesis by gene-encoded enzymes reconstituted in liposomes. Plos One.

[13] Kurihara K (2011). Self-reproduction of supramolecular giant vesicles combined with the amplification of encapsulated. DNA. Nat. Chem..

[14] Kurihara K (2015). A recursive vesicle-based model protocell with a primitive model cell cycle. Nat. Commun..

[15] Israelachvili, J. N. *Intermolecular and surface forces*. ch.20, pp.538–550 (Academic Press, 2011).

[16] Ringsdorf H, Schlarb B, Venzmer J (1988). Molecular architecture and function of polymeric oriented systems: Models for the study of organization, surface recognition, and dynamics of biomembranes. Angew. Chem. Int. Ed..

[17] Barton P, Hunter CA, Potter TJ, Webb SJ, Williams NH (2002). Transmembrane signalling. Angew. Chem. Int. Ed..

[18] Yamashita Y, Oka M, Tanaka T, Yamazaki M (2002). A new method for the preparation of giant liposomes in high salt concentrations and growth of protein microcrystals in them. Biochim. Biophys. Acta.

[19] Lerman LS (1971). A transition to a compact form of DNA in polymer solutions. Proc. Nat. Acad. Sci. USA.

[20] Kawakita H (2009). Formation of globules and aggregates of DNA chains in DNA/polyethylene glycol/monovalent salt aqueous solutions. J. Chem. Phys..

[21] Shohda K (2011). Compartment size dependence of performance of polymerase chain reaction inside giant vesicle. Soft Matter.

[22] Marsh D, Bartucci R, Sportelli L (2003). Lipid membranes with grafted polymers: physicochemical aspects. Biochim. Biophys. Acta.

[23] Toyota T (2008). Population study of sizes and components of self-reproducing giant multilamellar vesicles. Langmuir.

[24] Kurihara K, Takakura K, Suzuki K, Toyota T, Sugawara T (2010). Cell-sorting of robust self-reproducing giant vesicles tolerant to a highly ionic medium. Soft Matter.

[25] Matko J, Ohki K, Edidin M (1992). Luminescence quenching by nitroxide spin labels in aqueous solution. Biochem..

[26] Nomikos M (2007). Binding of phosphoinositide-specific phospholipase C-ζ (PLC-ζ) to phospholipid membranes. J. Biol. Chem..

[27] Gambhir A (2004). Electrostatic sequestration of PIP2 on phospholipid membranes by basic/aromatic regions of proteins. Biophys. J..

[28] Van der Meer, B. W., Coker, G. III & Chen, S.Y. S. *Resonance energy transfer: Theory and data* (Wiley-VCH, 1994).

[29] Schwartz E, Le Gac S, Cornelissen JJLM, Nolte RJM, Rowan AE (2010). Macromolecular multi-chromophoric scaffolding. Chem. Soc. Rev..

[30] Kamat PN, Tobé S, Hill IT, Szostak JW (2015). Electrostatic localization of RNA to protocell membranes by cationic hydrophobic peptide. Angew. Chem. Int. Ed..

[31] Meisel JW, Gokel GW (2016). A simplified direct lipid mixing lipoplex preparation: Comparison of liposomal-, dimethylsulfoxide-, and ethanol-based methods. Sci. Rep..

[32] Takakura K, Sugawara T (2004). Membrane dynamics of a myelin-like giant multilamellar vesicle. Langmuir.

[33] Breslow R, Dong SD (1998). Biomimetic reactions catalyzed by cyclodextrins and their derivatives. Chem. Rev..

[34] Kruger K (1982). Self-splicing RNA: Autoexcision and autocyclization of the ribosomal RNA intervening sequence of tetrahymena. Cell.

[35] Cech TR, Bass BL (1986). Biological catalysis by. RNA. Ann. Rev. Biochem..

[36] Breaker RR, Joyce GF (1995). A DNA enzyme with Mg^2+^-dependent RNA phosphoesterase activity. Chem. Biol..

