# Supplementary material for: DNA Length-dependent Division of a Giant Vesicle-based Model Protocell
Source: Sci Rep. 2019 May 6;9:6916. doi: 10.1038/s41598-019-43367-4 (PMC6502804; doi:10.1038/s41598-019-43367-4)
Supplement: Supplementary file 1 — Supplementary Information [file 41598_2019_43367_MOESM1_ESM.pdf]

Supplementary Information

DNA Length-dependent Division of a Giant Vesicle-based Model Protocell

M. Matsuo, Y. Kan, K. Kurihara, T. Jimbo, M. Imai, T. Toyota*, Y. Hirata, K. Suzuki & T. Sugawara*

Index

Supplementary Notes

1. Modification of vesicular membrane by adding PEG-grafted phospholipid

2. Deformation manner of a single PEG-grafted GV containing 1164-bp DNA

3. Preparation of DNA with different length and sequence

4. Composition of PCR solutions for PEG-grafted GVs containing different length of DNA

5. Size distribution of PEG-grafted GVs induced by the addition of **V***

6. Evaluation of the increase ratios of PEG-grafted GVs after the addition of **V***

7. Single GV observation after the addition of **V***

8. Dependence of increase ratio of GVs on DNA sequence

9. Fluorescence images of DNA-Texas Red in GVs containing TEMPOL in water phase

10. FRET experiments of GVs containing C-BODIPY and DNA-Texas Red

11. FRET detection in PEG-grafted GVs

12. UV-Vis spectral trace of decay of membrane precursor **V***

13. Cooperative mechanism of **V*** hydrolysis by lipo-deoxyribozyme consisting of DNA and amphiphilic catalyst **C**

Supplementary References

Supplementary Notes

1. Modification of vesicular membrane by adding PEG-grafted phospholipid

Polyethylene glycol (PEG) grafted on phospholipids in a vesicular membrane is thought to form mushroom structures at the interface between a water phase dissolving DNA and the membrane [1], suppressing the interaction between the DNA and the membrane. The interaction between longer DNA and the membrane is more suppressed by PEG-grafted phospholipid, compared with medium and short DNA (Figure S1). Under such a circumstance, there must be a suitable chain length of DNA to form the complex with cationic catalyst **C** dissolving in the PEG-grafted membrane.

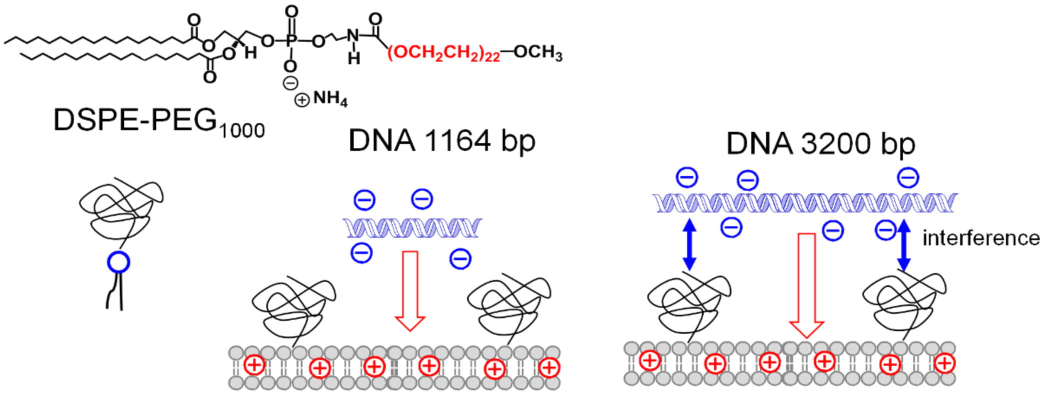

Figure S1. Schematic illustration of adhesion of middle or long DNA to PEG-grafted vesicular membrane.

Transformation of PEG chain from a mushroom to a brush regime is determined by the steric factor of the chain. The transition concentration, that is, the molar ratio of the amphiphilic macromolecular chain is expressed as equation S1 [1]:

$$X_n^{m\to b} = \frac{A}{\pi R_F^2} = \left(\frac{A}{\pi l^2}\right) n^{-6/5} \times 100 \quad [\text{mol\%}] \qquad \text{(S1)}$$

,where R_F is Flory radius of a PEG chain in water, A is a head group area of an amphiphile, l is a segment length and n is a degree of polymerization of the PEG chain. In this study, we used PEG1000- and 5000- grafted phospholipid to modify a vesicular membrane. Using an equation S1 for n = 22, l = 0.39 [nm], A = 0.69 [nm^2] [2, 3], the transition concentration of PEG1000 was calculated:

$$X_{22}^{\mathrm{m}\to\mathrm{b}} = 3.5 \text{ [mol\%]} \qquad \text{(S2)}$$

Similarly, the transition concentration of PEG5000 (n = 113) was calculated:

$$X_{113}^{\mathrm{m}\to\mathrm{b}} = 0.5 \text{ [mol\%]} \qquad \text{(S3)}$$

In order to evaluate the state of the PEG chain on a vesicular membrane surface, the coverage θ of PEG chain was calculated. The coverage θ [%] is given by:

$$\theta = \frac{n\pi R_{\mathrm{F}}^2}{S} \times 100 \qquad \text{(S4)}$$

,where R_F is Flory radius of the PEG chain, S is the surface area of GV, and n is the number of PEG-grafted lipids in GV. The vesicular surface area S is also expressed as equation S5:

$$S = \frac{100nA}{M} \qquad \text{(S5)}$$

,where A is the average area of a head group of phospholipid, and M [%] is the molar fraction of PEG-grafted lipid. Then, by comparing above equation S4 and S5, the expression for the coverage θ [%] follows as:

$$\theta = \frac{\pi M R_{\mathrm{F}}^2}{A} \text{ [\%]} \qquad \text{(S6)}$$

Thus, θ of PEG5000-lipid is estimated at 172% (for M = 0.85mol%. R_F = 6.7 nm, A = 0.69 nm^2), and θ of PEG1000-lipid is estimated at 24% (for M = 0.85mol%. R_F = 2.5 nm, A = 0.69 nm^2).

2. Deformation manner of a single PEG-grafted GV containing 1164-bp DNA

A single PEG1000 or PEG5000-grafted GV containing 1164-bp DNA with a diameter of not less than 5 μm and with thin lamellar was observed under a high-speed confocal laser scanning fluorescence microscope. Morphological changes of 30-40 PCR-subjected GVs stained by Texas Red-DHPE (0.2 mol%) were traced during 40 min after the addition of **V***. The deformation manner of each GV are summarized in Table S1.

Table S1. Deformation manner of PEG-grafted GVs during 40 min after the addition of V*

PEG-grafted lipid	DSPE-PEG1000	DSPE-PEG5000
Coverage	24%	172 %
Structure of PEG chain	Mushroom	Soft brush
Frequency of GV deformation		
Budding	40% (16/42) #	5% (2/32)
Nesting	55% (24/42)	95% (30/32)
No deformation	5% (2/42)	0% (0/32)

#) number of deformed GVs with corresponding manner / total number of deformed GVs.

3. Preparation of DNA with different length and sequence

In this study, five different DNA excised from pBR 322 vector; these lengths of DNA were 374 bp (short) 1164 bp (middle), 3200 bp (long) 1192 bp, and 1137 bp, (DNA sequences are shown in Figure S2). These DNA were amplified using primer sets (primers 1 and 2), (primers 1 and 3), (primers 1 and 4), (primers 5 and 6) and (primers 7 and 4), respectively (primer sequences are shown in Supplementary Method 2). 1164 bp-DNA has

almost the same chain length but no common parts of the sequence to 1192 bp and 1137 bp-DNA. On the other hands, 374 bp, 1164 bp and 3200 bp-DNA have a common sequence to each other (200-573).

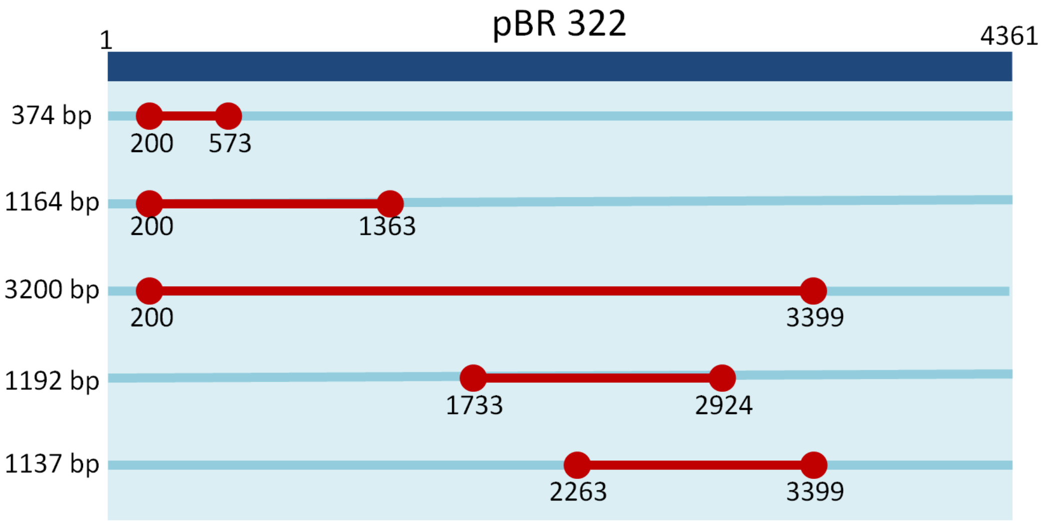

Figure S2. Sequences of DNA excised from pBR322 vector.

Random sequence of 1164 bp-DNA was generated as follows. The sequence of both ends for recognition sites of forward and reverse primers were the same as the used medium DNA (1164 bp) derived from pBR 322 vector in the previous section. The rest of the sequence was prepared according to the Python's random module mounting a Mersenne twister which was the pseudorandom number generator. Python's code is shown below.

```
import random
a = ['A','T','G','C']
length = [1124]
DNA = ''
f = open('DNA.txt', 'a')
for num in length:
for i in range(num):
DNA += a[random.randint(0,3)]
```

```
f.write(str(num) + ':' + DNA + '¥n')
DNA = ''
f.close()
```

The generated sequence of random DNA is shown below. Synthesized random DNA was purchased from Fasmac Co., Ltd. (Kanagawa, Japan), amplified by PCR and purified before the preparation of model protocells.

5’-GACAGCATCGCCAGTCACTAAGTCATGCACTTTGATAACAGCTCTCTAACTAACGCACCGGGAT

AGCACTTTATCCACGCTGGGTCGGTAGCATAGAAGGTCATCAACAGTAAAAGCTACCCGGGGCCT

ACAAAGCCATAGGGAGGCGGATAAGTGTGTGGTAGCAGTCTCGCCAACGACCGTTCACCTTCGGT

CTTTTTATGTGCTCGGCCTGTTCGTCGCGTTGAGAATGTATCCTATTCTAACCTTACCTTTGGGCATG

ATCCTCGCATGGCCTGGAGCTGGGATTGCACTGTATGCGCATTCTAGCCTGCTTTTGTAATGAGCTG

TAACTTAGTTGCCAAGACACAATTTTGGCATGGACACTTAGAGGAAGTAATAATCAATGTTCTTTA

AGGGTTCAAATCCGAACGGACTGCTCTTTTGCGCCAGGTGGCAAGTTTTTTCAATGAAAATTGCC

CGGGCAGTATCAAGCGATCCACCGAATTGCAGGAGGGCAGCTGCATCAATTGACCTCGGATAGTG

GGGACTAGTTCTCCGCTGGGTGATCAAGTACGAGTGTTGTGTGCCATAGCCTCGGGTCGGATCAAT

TAGCGGGTCACCCCGCGCAACATAGTTGAAATACCCCCGCCGGGCGAGGGGTAATAATGACCAAC

GTTGCTAATCAAGGGGGACAATCACTAAACTTAGGGCACGTGCACCGTATTGGCCCGTAGAATAAT

AATGGGCAAGACGTTTACTAGGTACGTCCCTTTCTTCTACGATCGTATACAGGAGCTGAGTAAAGC

ACAGGTGCATATTTAGGGCACATTCCGAATCTCCGAGCCCCTAGTGGGGACTAGAAAGGTCTTCGC

GCCCGTTTTGATCGCGTTATGGTCCACGAATTACATTTCACCCCCCCAAACATATGCGGCAGGACA

GGAGTGCAAAAATTCAACTTGGATTCCGGGCTTAGGGTGTCCAGCCAGACTGACACCGAGGGGT

AGAAATCATGAGTTCCGGCACTGTACCGCCTCGAACCTTCATCGCGGCCGCGTGCCGGGATACATC

TCTAAGACGCTGCACGAGCTTCAAAAGAAACCGAAGCGCCTCCTATCCTTCTGACAATAGAGTAC CTTACCCTAGCCGGTGCTCAGGCACAATCGAGAACTGTGAATGCGCAAA-3’.

4. Composition of PCR solutions for PEG-grafted GVs containing different length of DNA

The amounts of primers for GVs containing different DNA (374 bp, 1164 bp and 3200 bp) were adjusted because numbers of DNA was in inverse proportion to the length of DNA under the condition of the constant amounts of dNTPs in GV. The composition of PCR solution is shown and adjusted compositions are written in bold letters in Table S2.

Table S2. Composition of PCR solution between GVs containing three kinds of DNA (374 bp, 1164 bp and 3200 bp).

Aqueous solutions (concentration)	374 bp	1164 bp	3200 bp
Deionized water	289 µL	347 µL	365 µL
KOD buffer × 10	50 µL	50 µL	50 µL
$MgSO_4$ aq. (25 mM)	20 µL	20 µL	20 µL
dNTPs aq. (2 mM × 4)	40 µL	40 µL	40 µL
Forward primer aq. (10 µM)	**43 µL**	**14 µL**	**5 µL**
Reverse primer aq. (10 µM)	**43 µL**	**14 µL**	**5 µL**
Template DNA aq. (10 nM)	5 µL	5 µL	5 µL
KOD plus (DNA polymerase, 1unit /µL)	10 µL	10 µL	10 µL

5. Size distribution of PEG-grafted GVs induced by the addition of V*

In the flow cytometric measurement, a size of GV was estimated from the width of the front side scattering. We found that a diameter of polystyrene beads was directly proportional to the width of the front side scattering (FSC-W) in a range of 1 μm to 18 μm regardless of whether beads are stained by fluorescent probes or not (Figure S3). From the approximate straight line in Figure S3, a particle diameter *D* is given by:

$$D = \frac{FSC\text{-}W - 148}{13.8} \qquad \cdots (S7)$$

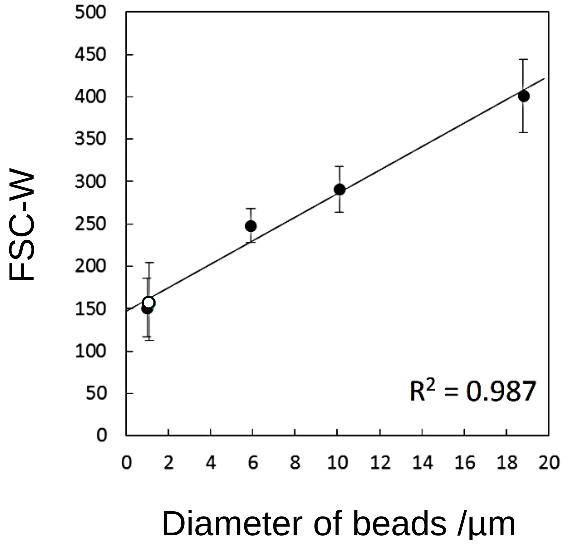

Figure S3. Correlation between the FSC-W and the particle size of polystyrene beads not stained (black dots) and stained with fluorescence probe (an open circle dot). Error bars are standard deviation. R is a correlation coefficient.

GVs with a diameter not less than 5 μm were selected by using equation S7, and original peaks and new peaks clearly differentiated in the fluorescence intensity histogram with the standing period for 48 h (30 min) in Figure 2 b-d in the text. The ratio of the remaining original peak to the new generated peak was shown in Table S3.

Table S3. Population analysis of fluorescence intensities of GVs containing different DNA measured before V* addition and 40 min after V* addition.

		Original	New Peak	Sample number, *N*
347 bp	before **V*** addition	1.0	—	1960
	30 min after PCR	0.05	0.95	680
	48 h after PCR	0.05	0.95	652
1164 bp	before **V*** addition	1.0	—	1677
	30 min after PCR	0.25	0.75	1180
	48 h after PCR	0.25	0.75	2074
3200 bp	before **V*** addition	1.0	—	2472
	30 min after PCR	0.40	0.60	1934
	48 h after PCR	0.33	0.67	2357

N represents the total number of GVs with the diameter not less than 5 μm. Total numbers of detected GVs were 10^4.

The value of FSC-W, which corresponded to the sizes of GVs with the standing period for 48 h, were measured before and at 40 min after the addition of **V***. The FSC-W distributions of three kinds of GVs (including GVs with diameter less than 5 μm) are shown in Figure S4. The original distribution was the same to all three types of GVs, indicating that the length of encapsulated DNA exerted no influence on the size of three kinds of GVs.

When the population of GVs (S) was measured at 40 min after the addition of **V***, the population of large GVs in the original distribution mostly decreased, and a new distribution centred at ca. 2.5 μm appeared. In the

histogram of GVs (M) at 40 min after the addition of **V***, the peak position which centred at 4 μm was almost same as before the addition of **V*** in spite of the peak position of fluorescence intensity became half (Figure 2c in the text). It means that the size of GVs (M) restored at 40 min after the addition of **V***. In the case of GVs (L), a similar change to that of GVs (M) was observed in the histogram, but a new peak assignable to smaller GVs was much suppressed. The smaller degree of the change was ascribed to the slow rate of deformation of GVs (L). Although the major peaks of GV (M) and GV (L) with standing period for 48 h centred at 4 μm appeared after the addition of **V***, the centre of the peak of GV (S) was observed at 2.5 μm. It means that a GV (S) tends to divide into small GVs than GVs (M) and (L).

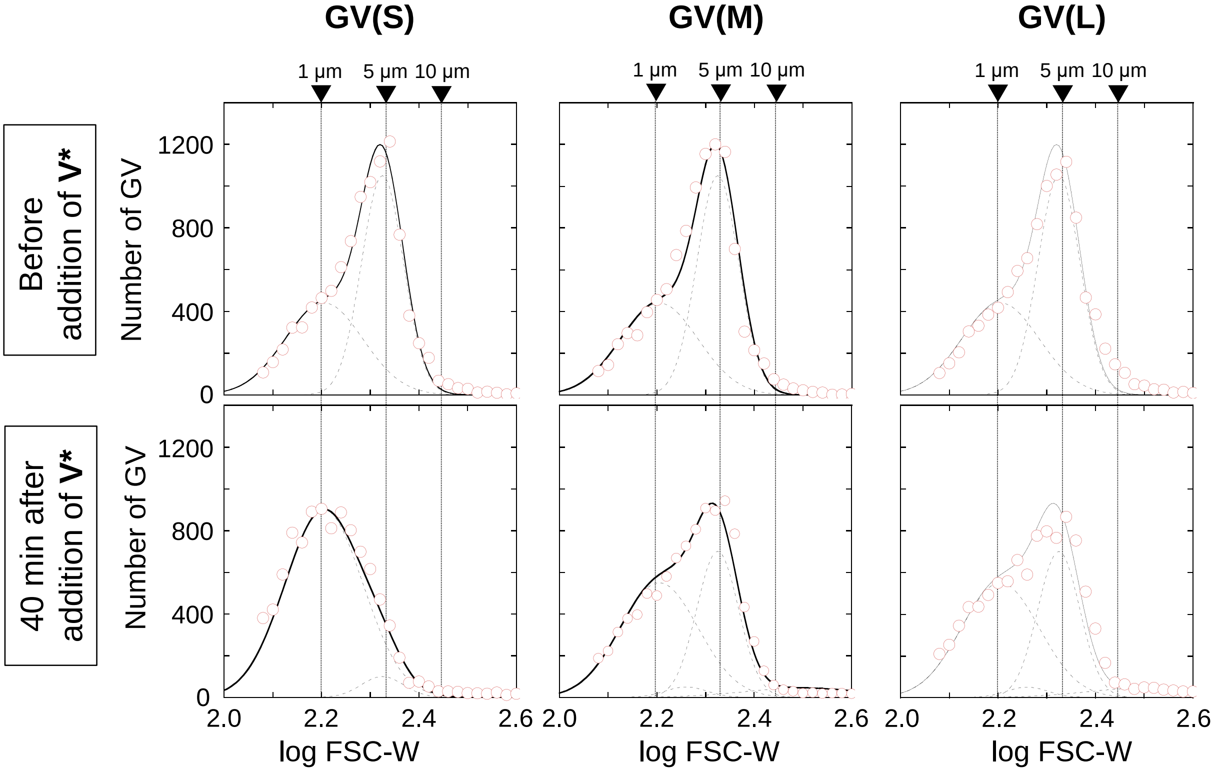

Figure S4. Histogram of FSC-W in PEG-grafted GVs containing different DNA 40 min after the addition of V* with standing period for 48 h. after PCR. The profiles of histogram were decovoluted by a couple of Gaussian curves (dotted lines) and fitted by the summation of Gaussian curves (blue lines).

6. Evaluation of the increase ratios of PEG-grafted GVs after the addition of V*

A dispersion of GVs was placed into a frame-chamber on a glass plate with cover glass. A counting method of GVs in the frame-chamber which was put on a confocal laser-scanning fluorescence microscope was illustrated in Figure S5. A number of GVs in five observation fields of 1350 μm × 1350 μm (4 corners and one in the middle) in the frame-chamber was captured as a sliced wide image by a confocal microscope, and all GVs on 25 sliced images along the depth direction were counted by viewer and analyser software.

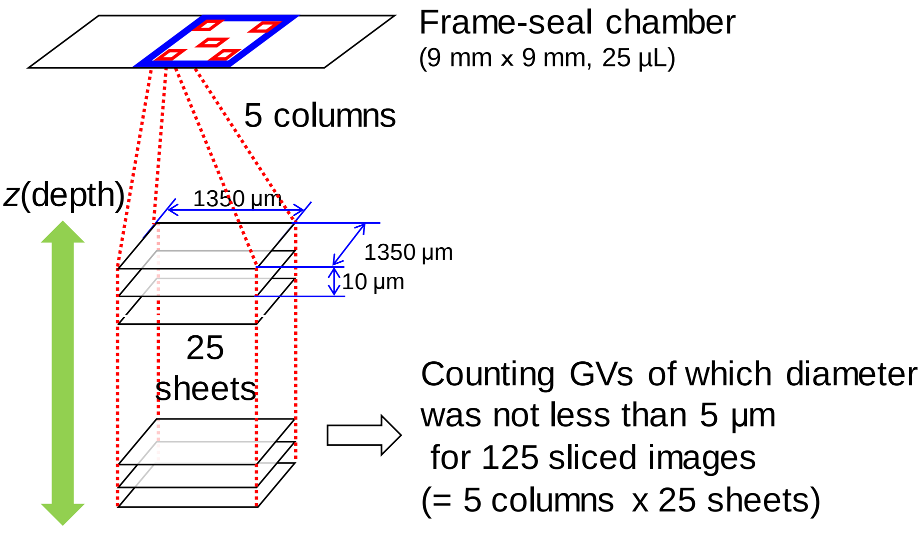

Figure S5. Schematic diagram of counting the number of GVs by a confocal laser scanning fluorescence microscope.

The numbers of GVs (S), (M), and (L) were counted before the addition of **V*** (0 min) and at 30 min and 60 min (incubation period) after the **V***addition. The data are plotted in Figure S6.

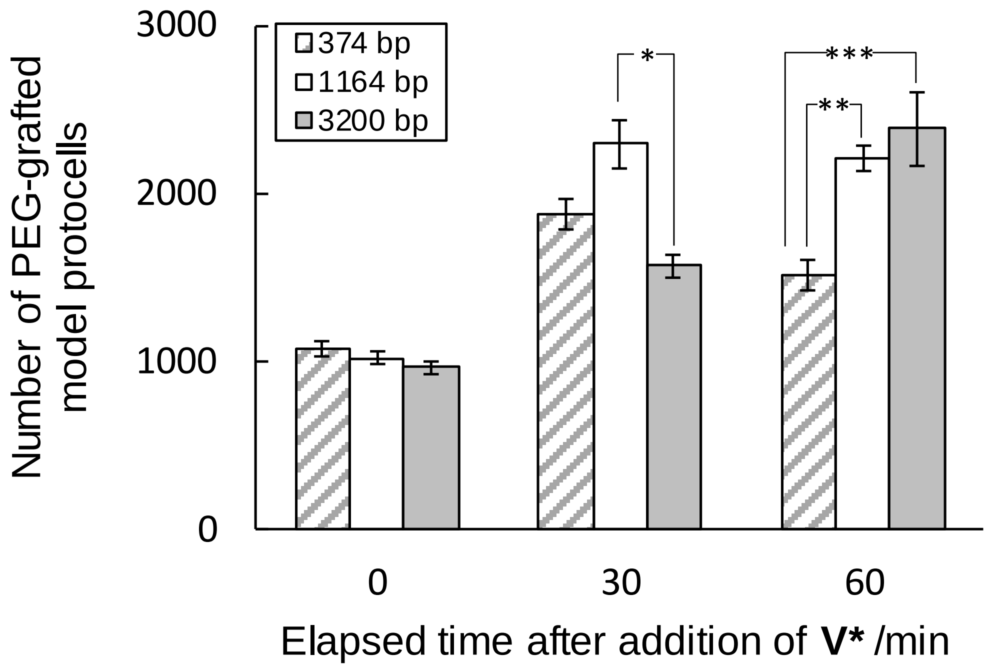

Figure S6. Numbers of PEG-grafted GVs before and after the addition of V*. Increased numbers of GVs containing different DNA showed differences at 5 % significance level. *) p = 0.02, **) p = 0.02, ***) p = 0.02. Error bars represent standard errors.

7. Single GV observation after the addition of V*

Figure S7 shows a CLMS image of the dispersion of the PEG-grafted GVs containing 1164 bp-DNA 30 min after the addition of **V***.

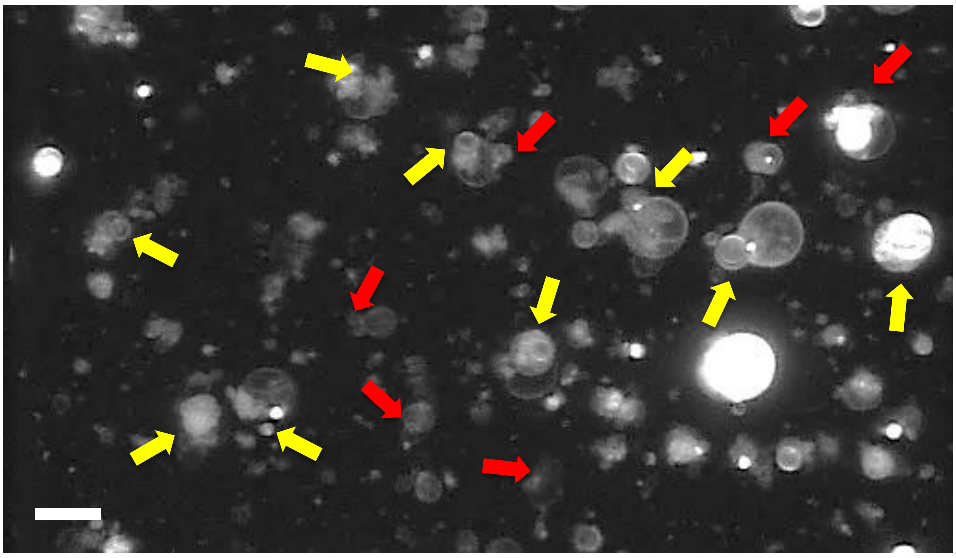

Figure S7. A confocal microscope image of the PEG-grafted GVs containing 1164 bp-DNA at 30 min after the addition of V*. Yellow arrows indicate nesting deformations and red arrows indicate budding deformations

Deformation manners of a single GV(S), (M), and (L) stained by Texas Red-DHPE (0.2 mol%) were traced for 40 min using 30-40 PCR-subjected GVs (a diameter of not less than 5 μm and with thin lamellar) after addition of **V*** by a confocal microscope. The dependence of deformation manners of a single PEG-grafted GVs were summarized in Table S4.

Table S4. Dependence of deformation manner of single PEG-grafted GV on the length of DNA.

DNA length	374 bp	1164 bp	3200 bp
Frequency of deformation			
Budding			
Equivolume	0% (0/38) [#]	38% (16/42)	9% (3/33)
Non equivolume	34% (13/38)	0% (0/42)	18% (6/33)
Nesting	66% (25/38)	57% (24/42)	55% (18/33)
No deformation	0% (0/38)	5% (2/42)	18% (6/33)

[#]) number of deformed GVs with corresponding manner / total number of deformed GVs. The definition of the "equivolume budding" is the diameter of the daughter GVs (region) is larger than 70% of the original GV.

Angelova *et al.* reported that when aliquots of aqueous solutions containing DNA of different lengths (21

bases, 250 bp, or 5.6 kbp) were applied with a micro-injector to the surface of GVs containing the cationic amphiphile *D*-sphingosine, the morphological changes occurred depending on the length of the encapsulated DNA [4]. This result shows that the manners of deformations of GVs could be influenced by the DNA of different lengths, although the purpose of this experiment is not directly related to the current investigation which is focused on the division dynamics of GVs.

8. Dependence of increase ratio of GVs on DNA sequence

The increased numbers of four kinds of GVs containing different DNA (1164 bp, 1137 bp, 1192 bp and random 1164 bp) in which the lengths of DNA are almost same but the sequences are differentiated. The sliced images of GVs were captured by a confocal microscope (Eclipse Ti, Nikon, Tokyo, Japan) before and 45 min after the addition of **V*** (the standing period was 48 h). Numbers of GVs before and after the **V*** addition were obtained by counting all the sliced images. However, significant differences were not found in the increase ratios between GVs containing four kinds of DNA as shown in Table S5.

Table S5. Increase ratios were calculated between GVs containing four kinds of DNA (1164 bp, 1137 bp, 1192 bp and random 1164 bp).

DNA Type	Increase ratio of PEG-grafted GVs 45 min after addition of V*
1164 bp	2.39 ± 0.14
1137 bp	2.71 ± 0.07
1192 bp	2.55 ± 0.19
random 1164 bp	2.54 ± 0.07

9. Fluorescence images of DNA-Texas Red in GVs containing TEMPOL in water phase

Confocal microscopy images (via Texas Red channel) of the GVs prepared in absence of TEMPOL and in presence of 500 mM TEMPOL (Figure S8): TEMPOL works as a water soluble quencher of Texas Red. As shown in Figure S8a, the DNA-Texas Red emitted from both the water phase and the membrane in the absence of TEMPOL. While the FL intensity of DNA-Texas Red from the water phase was distinctly suppressed in the presence of TEMPOL, the FL from the membrane was intact. It means that DNA-Texas Red remained in close physical proximity of the membrane.

a)

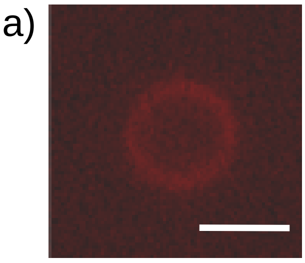

b)

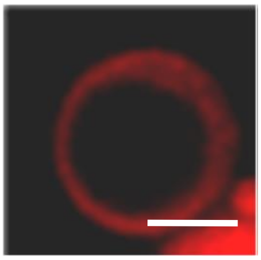

Figure S8. Fluorescence images of DNA-Texas Red in GV containing / not containing TEMPOL in water phase. Confocal microscopy images (via Texas Red channel) of the GVs prepared in absence of TEMPOL (a), and in presence of 500 mM TEMPOL (b). Both dispersion of GVs were incubated for 51 h after PCR. Texas Red emission from membrane was detected regardless of the presence of TEMPOL. Scale bars represent 5 μm.

10. FRET experiments of GVs containing C-BODIPY and DNA-Texas Red

Confocal microscopy images of the GVs containing only **C**-BODIPY observed through a donor channel (Figure S9 a), only DNA-Texas Red observed through an acceptor channel (Figure S9 b), and both probes observed through a FRET channel (Figure S9 c). GVs were incubated for 51 h after PCR. Scale bars represent

5 μm. Leakage of fluorescence into each channel was not observed under the current condition.

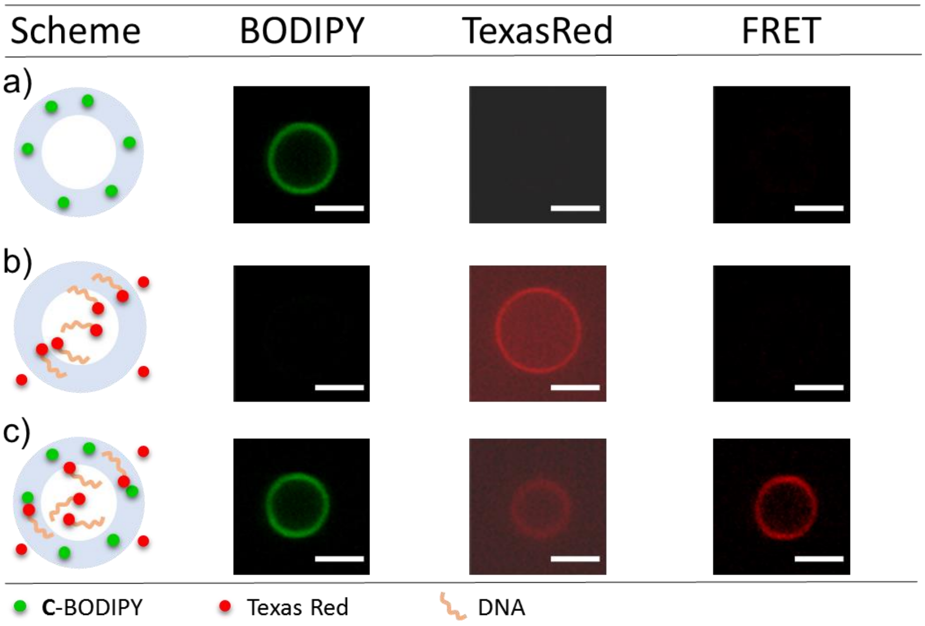

Figure S9. Confocal microscopy images of PCR-subjected GVs containing only C-BODIPY (a), only DNA-Texas Red (b) and both probes (c). Scale bars represent 5 μm.

Averaged fluorescence intensities of BODIPY over several samples emitted from the membranes of GVs (at 51 h after PCR) containing different length DNA observed through the donor channel, and those of Texas Red through the acceptor channel were shown in Figure S10: GVs involved 20 bp, 374 bp or 1164 bp-DNA, respectively. No significant differences in the DNA chain-dependence of BODIPY intensities and of Texas Red intensities were found (Figure S10). On the other hand, the FRET emission clearly observed through a FRET channel was shown in Figure 5a in the text and it showed significant differences between FRET intensities of GVs containing different DNA (Figure 5 in the text).

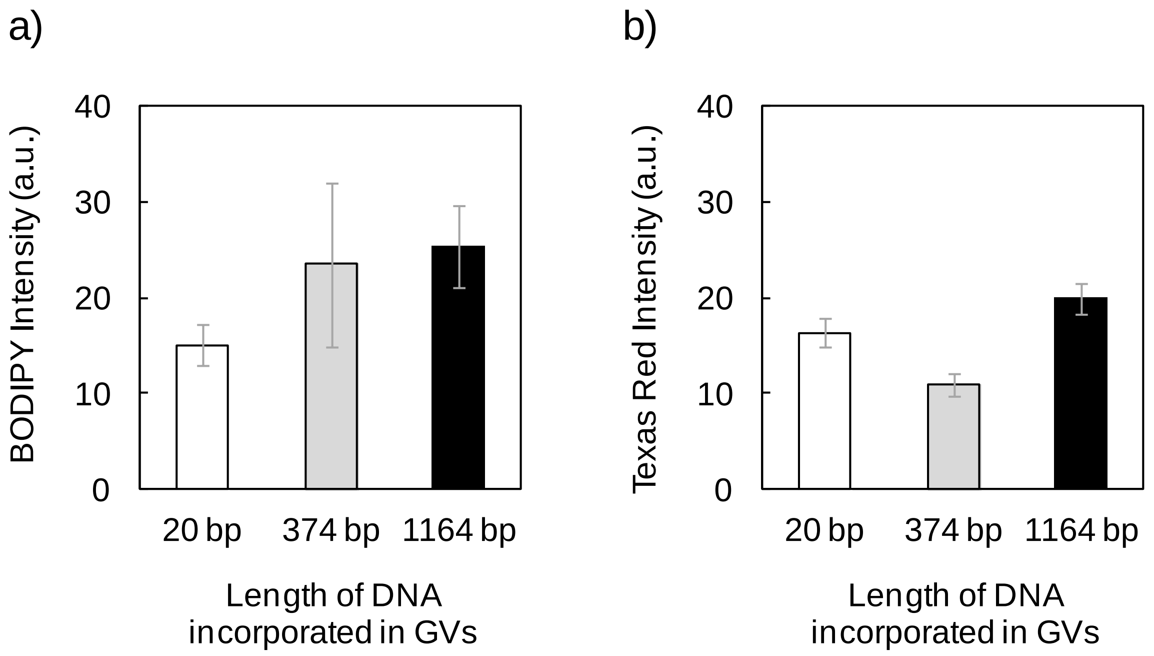

Figure S10. Fluorescence intensities of BODIPY and Texas Red in GV-based model protocell containing different DNA lengths. Average fluorescence intensities of BODIPY channel (a) and Texas Red channel (b) emitted from the membrane of GVs. GVs incorporated 20 bp (*n* = 6), 374 bp (*n* = 3) or 1164 bp (*n* = 5) DNA, respectively, at 51 h after PCR. Error bars represent the standard errors.

11. FRET detection in PEG-grafted GVs

Confocal microscopy images of PEG-grafted GVs containing both **C**-BODIPY and DNA-Texas Red probes were captured 51 h after PCR treatment. FRET intensities were observed even from the membrane of PEG-grafted GVs regardless of the presence of PEG-grafted phospholipid (Figure S11).

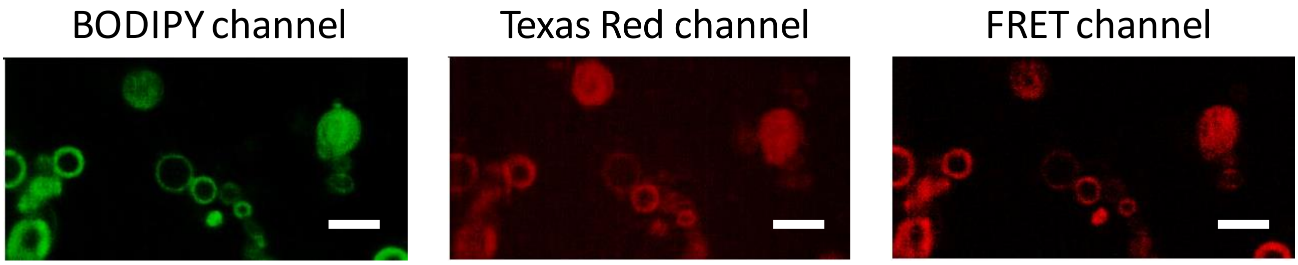

Figure S11. Confocal laser scanning fluorescence microscopy images of PEG-grafted GVs containing both C-BODIPY and DNA-Texas Red probes. Scale bars represent 10 μm.

12. UV-Vis spectral trace of decay of membrane precursor V*

Time course change of UV-Vis spectra during the decay of membrane precursor **V*** after addition of **V*** solution (156 μM) to the dispersion of aggregates composed of DNA and catalyst **C**. This procedure was chosen to avoid monitoring light-scattering due to the presence of GVs in the dispersion. The absorbance intensity at 332 nm was assigned to the benzylidene aniline and monitored every 5 minutes at room temperature (Figure S12) [5].

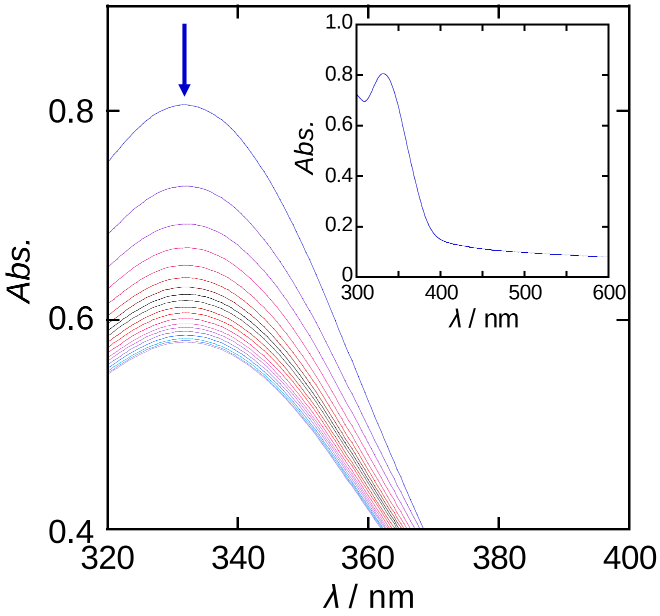

Figure S12. UV-Vis spectral trace of decay of membrane precursor V*.

13. Cooperative mechanism of V* hydrolysis by lipo-deoxyribozyme consisting of dsDNA and amphiphilic catalyst C.

The enhanced catalytic activity of amphiphilic catalyst **C** carrying a hydrochloride salt of the imidazolium derivative as a hydrophilic group may be explained by the cooperative dynamics between two adjacent catalysts. Breslow synthesized cyclodextrin bis(imidazoles), in which one of the imidazolium groups was protonated, and demonstrated a synergistic effect of these two groups on the hydrolysis of esters tapped in the cavity. He claimed that it worked as an artificial enzyme [6]. In our study, FRET experiments revealed that **V** and **C** are cationic and also combined to DNA due to electrostatic interaction (Figure S13). According to Breslow's study, the plausible cooperative mechanism of DNA and **C** would be as follows; **V*** is sandwiched by two imidazolium groups of **C**, a part of which may be deprotonated in buffer (pH 8.0) because of the weak acidity (pKa = 6.9) of the protonated imidazolium group. The difference between pH of the buffer and the pKa of the imidazolium salt seems to be too large, but if pH in a lipid membrane is compared with that in aqueous solution, the former is sufficiently smaller as discussed in the literature [7]. This interpretation needs further proofs which would be obtained by spectrometric studies.

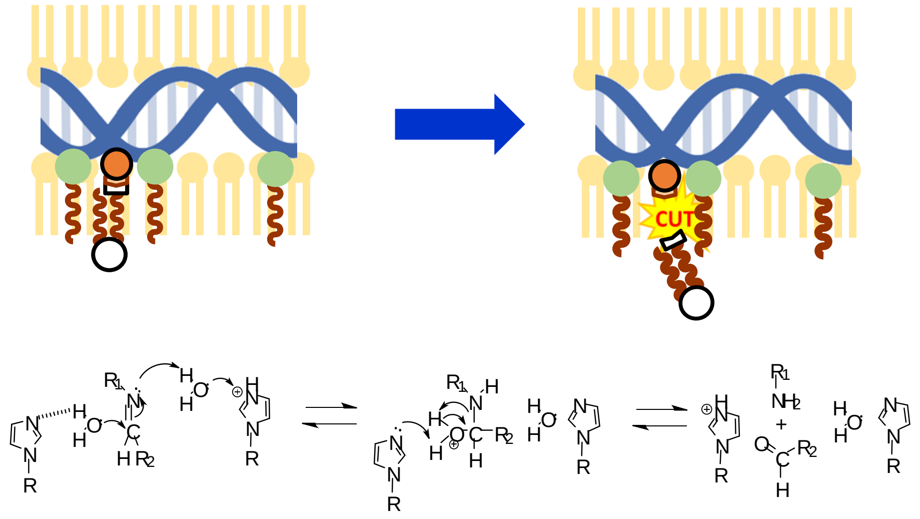

Figure S13. Schematic structure of lipo-deoxyribozyme.

Supplementary References

1. Marsh, D., Bartucci, R. & Sportelli, L. Lipid membranes with grafted polymers: physicochemical aspects. *Biochim. Biophys. Acta* **1615,** 33-59 (2003).

2. Vacha, R., *et al.* Effects of alkali cations and halide anions on the DOPC lipid membrane. *J. Phys. Chem. A* **113**, 7235-7243 (2009).

3. Nagel, J. F. & Tristran-Nagle S. Structure of lipid bilayers. *Biochim. Biophys. Acta* **1496**, 159-195 (2000).

4. Angelova, M. I. & Tsoneva, I. Interactions of DNA with giant liposomes. *Chem. Phys. Lipids* **101,** 123-137 (1999).

5. Takakura, T. & Sugawara, T. Membrane dynamics of a myelin-like giant multilamellar vesicle applicable to a self-reproducing system. *Langmuir* **20,** 3832-3834 (2004).

6. Breslow, R. & Dong, S. D. Biomimetic reactions catalyzed by cyclodextrins and their derivatives. *Chem. Rev.* **98,** 1997-2012 (1998).

7. Ishimaru, M. Toyota, T. Takakura, K. Sugawara, T. & Sugawara, Y. Helical aggregate of oleic acid and its dynamics in water at pH 8. *Chem. Lett.* **34,** 46-47 (2005), and references cited therein.
